# Those who ignore the past are doomed…to be heartless: Lay historicist theory is associated with humane responses to the struggles and transgressions of others

**DOI:** 10.1371/journal.pone.0246882

**Published:** 2021-02-19

**Authors:** Michael J. Gill, Michael R. Andreychik, Phillip D. Getty

**Affiliations:** Department of Psychology, Lehigh University, Bethlehem, PA, United States of America; University of Klagenfurt, AUSTRIA

## Abstract

When one learns that current struggles or transgressions of an individual or group are rooted in an unfortunate history, one experiences compassion and reduced blame. Prior research has demonstrated this by having participants receive (or not) a concrete historicist narrative regarding the particular individual or group under consideration. Here, we take a different approach. We explore the possibility that everyday people show meaningful variation in a broad lay theory that we call *lay historicism*. Lay historicists believe that—as a general fact—*people’s psychological characteristics and life outcomes are powerfully molded by their life histories*. We present eight studies linking lay historicism to broad tendencies toward compassion and non-blaming. Collectively, Studies 1–5 suggest that lay historicism affects compassion and blame, respectively, via distinct mechanisms: (1) Lay historicism is associated with compassion because it creates a sense that—as a general fact—past suffering lies behind present difficulties, and (2) lay historicism is associated with blame mitigation because historicists reject the idea that—as a general fact—people freely and autonomously create their moral character. Thus, lay historicism increases compassion and decreases blame via distinct mechanisms. The remaining studies diversify our evidence base. Study 6 examines criminal justice philosophies rather than broad moral traits (as in the earlier studies) and shows that lay historicism is associated with preference for humane criminal justice philosophies. Study 7 moves from abstract beliefs to concrete situations and shows that lay historicism predicts reduced blaming of an irresponsible peer who is encountered face-to-face. One additional study—in our Supplemental Materials—shows that lay historicism predicts lower levels of blaming on implicit measures, although only among those who also reject lay controllability theories. Overall, these studies provide consistent support for the possibility that lay historicism is broadly associated with humane responding to the struggles and transgressions of others.

## Introduction

If we could read the secret history of our enemies, we should find in each [person’s] life sorrow and suffering enough to disarm all hostility.

—Henry Wadsworth Longfellow

*None of us could predict that if we had been subjected to the same influences*, *the same conditioning*, *we would not have turned out like these perpetrators*.

—Archbishop Desmond Tutu

Psychological scientists have devoted much effort to understanding the psychological factors that promote prosocial responses such as compassion and helping, and the factors that temper antisocial responses such as hatred and harming. Numerous factors have been implicated in fostering these humane responses: Perspective taking [1, 2], shared identity [3, 4], moral rules or principles [5, 6], desire to avoid disappointing valued others [7, 8], and moral emotions [9, 10].

In the present article, we aim to add to the literature on the psychology of humane responses to others’ struggles and transgressions. Our conceptual focus has roots in one the most generative and enduring theories of social psychology—attribution theory—but we also offer a novel conceptual contribution and novel empirical findings. Hundreds of studies show that attributions for others’ behavior affect the extent to which we feel compassion for them and the extent to which we blame them harshly for their misdeeds [11–14]. In this extensive literature, attributions are directly provided by the researchers and are specific to the actor and behavior at hand. That is, researchers provide information that explains why *that person* engaged in *that action*.

Here, we focus on an alternative possibility: Individuals have their own *lay theories* of behavior causation, which they apply broadly across many different actors and behaviors. They do this without needing to be provided with any explanatory account by others. More specifically, our novel contribution is the idea that everyday people show meaningful variation in a heretofore unexamined lay theory that we call *lay historicism*. Lay historicists believe that, as a general fact about the social world, *people’s psychological characteristics and life outcomes are powerfully molded by their life histories*. That is, a lay historicist believes that a person’s life history explains why she is nice or mean, why he is devoted to children or abhors them, why she is a perfectionist or a slacker, why he joins a violent neo-Nazi group or a staid book club, and so on.

Prior work suggests that humane responses are increased when perceivers are explicitly guided to think about a target person’s life history by being provided with information about that history [12]. We will show that lay historicists show humane responses without needing such prompting. They do so because they habitually assume the presence and causal influence of explanatory life histories without needing to be specifically informed about them. Indeed, if everyone were a lay historicist, then the presentation of a historicist narrative would not have the very large effect that has been shown in experimental work. Apparently, then, although people are sensitive to the implications of historical information that is explicitly provided [12], this does not necessarily lead them to become a lay historicist who habitually assumes historical causation.

Although some prior work focuses on attributions of behavior to life history (and how such attributions are distinct from attributions to situational context; [12, 15–17]), and much prior work focuses on lay theories [18], there is no prior work suggesting the existence of a broad lay theory of historical causation which contributes broadly to compassion and non-blame. Our central hypothesis is that lay historicism fosters a broad tendency to respond humanely to others.

Below, we present eight studies to test this hypothesis. In most studies we will measure individual differences in lay historicism using Gill and Andreychik’s *Social Explanatory Styles Questionnaire* [15]. We will assess humane responding in a variety of ways: As broad dispositions (e.g., dispositional compassion proneness, dispositional outrage proneness), as social policy preferences (e.g., preference for harsh versus humane treatment of criminals), and as reactions to specific target individuals (e.g., blame of a peer encountered in a face-to-face interaction). We will provide evidence using both explicit and implicit measures. In several studies, we will include tests of mediation to illuminate *why* lay historicism promotes humane responses.

In short, this article will explore the possibility that those who habitually ignore the past—*non-historicists*—are doomed to be hard-hearted in their responses to others, whereas as those who naturally and spontaneously think about the past—*lay historicists*—will be relatively compassionate and devoid of harsh blame responses across a variety of indices. Before providing relevant evidence, we will situate our work within the existing literature.

## Attribution theory: The quest for causal understanding of behavior

Heider [19] offered an extensive exploration of how the drive for causal understanding operates in the social realm. He emphasized that people are continuously seeking causal understanding of why others act as they do and that they seek such understanding because it facilitates social adaptation. Heider inspired the development of additional theories that attempted to describe the reasoning processes people utilize to arrive at a “proper” explanation [20, 21]. This work famously characterized the social perceiver as a *naïve scientist* who uses logic to arrive at a proper causal explanation for observed behavior [22].

Within the naïve scientist framework, explanations were “dependent variables,” and experiments examined the conditions under which particular explanations would be selected. Weiner was influential in shifting explanations to the role of “independent variable,” and examining how explanations cause emotional (e.g., anger) and motivational (e.g., urge to harm) states within the explainer [14]. Furthermore, Weiner introduced a second shift: Social perceivers are not (only) naïve scientists, they are also moral actors motivated to assign blame and praise and to reward and punish accordingly. We will label this the *naïve moralist* perspective.

### From *locus* to *controllability*

Within the naïve scientist framework perceivers were assumed to be interested in deciding whether actions were caused by “internal” *dispositions* of the actor or by “external” features of the actor’s current *situation* [23]. Choosing between these two causal loci—internal vs. external—facilitates the naïve scientist’s goal of predicting a person’s future behavior. The reason is that dispositional explanations imply that an act will recur, whereas situational explanations imply that it will not.

Weiner’s work initiated a shift from the locus dimension of attribution to the *controllability* dimension, which refers whether an action is volitionally alterable by the actor. *Could the actor have chosen to do otherwise*? Generally, Weiner’s empirical work treats controllability as a dimension that varies within the “internal” side of the locus dimension. That is, controllability is present when actions reflect “choice” (“sin”) but diminished when actions reflect mechanisms such as biological abnormality (“sickness”) [14], both of which are internal to the actor. Attending to controllability facilitates the naïve moralist’s goal of moral evaluation. Specifically, a controllable explanation implies “free will” and hence blameworthiness, whereas an uncontrollable explanation implies determinism hence reduced blameworthiness.

### From *controllability* to *historicism* and two distinct types of control

Gill and his colleagues [12, 15–17] offered further conceptual development, building upon the frameworks just described. Specifically, Gill and Cerce [12] offered the concept of *historicist narratives*, which provide a story-like explanation of how an individual became who she is based on pivotal formative experiences. They showed that historicist narratives regarding transgressors temper blame despite having no impact on perceptions of the transgressor’s negative intentions, volitional control (i.e., Weiner’s controllability), or antisocial dispositions (i.e., internal locus). If historicist narratives do not affect any of these well-established contributors to blame, then how do they mitigate blame?

Gill and Cerce [12] proposed that everyday moralists utilize two distinct concepts of free will when assessing blameworthiness: *Freedom of action* (i.e., Weiner’s controllability) and *control of self-formation* (a novel concept). Whereas freedom of action refers to the extent to which an actor is seen as having the capacity to volitionally alter actions “in the present moment,” control of self-formation refers to the extent to which an actor is thought to have self-created her own enduring moral traits. Within this framework, a transgressor can be seen as having a hostile disposition that he never set out to have (i.e., limited control of self-formation), yet as retaining the ability to “control himself” and replace hostile actions with alternative actions in a given moment. For Gill and Cerce, then, freedom of action and control of self-formation represent two distinct notions of control or free will. Furthermore, their studies suggested that only the latter is relevant for understanding how historicist narratives temper blame and punishment.

## A broader perspective: Historicism as a lay theory

The present work is an extension of the theoretical progression just described. Here, we continue the focus on historicist thinking reflected in Gill and Cerce [12], but we explore the possibility that historicism exists as a broad lay theory. This differs from prior work in which concrete historicist explanations were explicitly presented as experimental manipulations: *Here is a story of how James became a bully*. The lay theory approach implies that concrete history information need not be explicitly provided to generate the prosocial impacts of historicist thinking. This is because lay historicists embrace a broad, abstract historicist theory which creates a broad, general tendency of humane responding across different targets and contexts. This general propensity for humaneness occurs because lay historicists spontaneously assume or impute an explanatory historicist narrative behind others’ actions and struggles.

Our lay theories approach is inspired by work in cultural psychology [24–26] and by work by Dweck and her colleagues [18, 27–29]. A lay theories approach “assumes that perceivers vary in their general beliefs and expectations regarding social causality” and tend to “see the world in terms of their theories” [15; pg. 2]. From this perspective, explanations are not generated in a bottom-up fashion on a case-by-case basis, but rather are generated in a top-down fashion, based on application of the perceiver’s pre-existing theories of social causality. Perceivers assume that, in any particular case, causality operates in the manner specified by their theories (“Of course his life history caused his mean personality; that’s how personality is formed.”).

Gill and Andreychik adopted the lay theories perspective championed by Dweck and her colleagues but offered a different conceptualization of the contents of those theories [15]. Here, we foreground Gill and Andreychik’s concept of lay historicist theories. Their Social Explanatory Styles Questionnaire (SESQ) measures lay historicism by presenting respondents with eight behaviors—four negative, four positive—performed by eight different individuals: e.g., *Sarah often ridicules and belittles her children*. *She tells them they are lazy*, *sloppy*, *and even “worthless*.*”* For each behavior, the respondent sees a “WHY?” question, with the wording changed to be suitable for the behavior: *WHY has Sarah become such a cruel mother*? Following the “WHY?” question is an item that taps agreement with a historicist explanation: *A major factor is Sarah’s prior life experiences*, *or personal life history*. Participants indicate their agreement on a five-point scale where “1” is labeled *NO* and “5” is labeled *YES*. Lay historicism is calculated by averaging across the eight ratings of the historicist explanation. See the S1 File for the full SESQ. This is the measure of lay historicism we will use in seven of our eight studies below.

Across six studies, Gill and Andreychik presented evidence for the psychometric soundness of the SESQ and for the predictive validity of each of its three subscales [15]. Crucially for present purposes, Gill and Andreychik found that lay historicism was unrelated to whether people were entity or incremental theorists (i.e., Dweck’s concept of lay theories; [18]), a finding that will be replicated below. Lay historicism was also unrelated to the implicit trait and implicit context theories of Church and colleagues [30]. Thus, in linking lay historicism to a broad tendency toward humane responding, we are providing a contribution that is distinct from existing work on lay theories.

## Historicism and humane responding

Why do we expect lay historicism to contribute to humane responding? This prediction is well-grounded in prior work. That prior work, however, has manipulated historicist explanations regarding particular targets rather than testing whether historicism exists and functions as a broad lay theory. Although prior work has not taken a lay theories approach, it has paid careful attention to mechanisms and thus provides guidance regarding appropriate hypotheses to explore in the present article.

### Historicism and compassion

Gill, Andreychik, and Getty were interested in understanding the factors that facilitate compassion toward outgroups [31]. They focused on the role of historicist explanations (they used the label “external,” which we reject because it conflates “situational” and “historical” [12]). Across four studies using several distinct target groups they found that historicist explanations promoted compassionate responses. Furthermore, they found strong evidence that the impact of historicist narratives on compassion traveled through *perceived suffering* of the outgroup. Thus, the evidence suggested that when people contemplate the role of history in shaping the struggles or misdeeds of an outgroup, they infer that the history must have been painful for the group, and this inference promotes the experience of compassion.

### Historicism and the tempering of harsh blame

Rather than focusing on compassion, other work has linked historicism to reduced outrage and punitiveness. For example, several studies have found that offenders who have endured a history of abuse are more likely to be treated mercifully (e.g., spared the death penalty) than are offenders who have not suffered abuse [32–36]. Gill and Cerce [12]—reviewed in detail above—similarly showed that historicist narratives reduced outrage and punitiveness and added that this occurs due to diminished perceptions of control of self-formation. They showed that, unlike for compassion, perceived suffering makes no difference in blame judgments. This evidence for distinct mechanisms linking historicism to compassion versus blame will form the basis for our hypotheses in several studies below.

## The present studies

### Ethics statement

All studies were approved by Lehigh University’s Institutional Review Board (Approval ID#s: 158373, 281949–5, 428016–1, 1149682–11). For in-person studies, participants provided written informed consent. For web-based studies, participants provide informed consent by clicking an "agree" button after reading the IRB-approved consent form.

### Overview of studies

Below, we present eight studies—including one in the S2 File—that explore the link between lay historicist theories and humane responding. Because our core construct is an individual difference variable, the studies are all correlational in nature. Although correlational studies cannot provide information regarding causality, the causal impact of historicist narratives—and of attributions and lay theories more broadly—is already beyond doubt [12, 14, 16–18, 31]. What is currently missing from the literature is evidence regarding historicism as a lay theory.

As noted in the Introduction, perceived controllability is a well-established contributor to blame [14]. Gill and Andreychik’s SESQ [15]—which we use to measure lay historicism—also includes a scale to tap lay controllability theories. For the studies described in this article, whenever possible (i.e., for 7 of our 8 studies; Study 5 did not use the SESQ) we tested the effects of lay historicism, lay controllability theories, and their interaction on the main dependent variable. Below, we describe all cases in which we found a main effect or interaction involving lay controllability theories. Notably, effects of lay controllability theories were only found when blame was the dependent variable and never for compassion.

Finally, all studies had ample statistical power. Please the S2 File for power analyses for each study.

## Study 1: Lay historicism, perceived suffering, and dispositional compassion proneness

Study 1 tests the idea that lay historicists will show heightened dispositional compassion proneness. *Compassion* is an emotion elicited by the perceived suffering or need of another. The subjective experience of compassion is described by words such as *sympathetic*, *tender*-*hearted*, *caring*, and *concerned*, and these feeling states create a motivation to improve the welfare of the other [37]. Self-reports of compassion, as we will use below, are predictive of prosocial behaviors [38]. Furthermore, we will test a mediational hypothesis suggested by prior work: The effect of lay historicism on compassion proneness travels through the perception that prior suffering lies behind people’s current bad actions or decisions.

### Method

#### Participants

Participants were 151 individuals (68 male) who received course credit in their introductory psychology course.

#### Procedure

After providing informed consent, participants completed our measures of *lay historicism* and *lay controllability theories*. These were measured using the SESQ [15], which was described in the Introduction and is available in the S1 File. In fact, for all studies in this article all scales and manipulations other than those published by other authors are available in the S1 File.

To measure *perceived suffering*, participants completed six items (e.g., *A major reason that a person is unkind is that s/he has had much emotional pain in life*) modeled after items in [31]. We averaged the six items to form an index of *perceived suffering*. Finally, to tap compassion proneness, participants completed the Compassionate Love Scale (CLS), which has been shown to predict prosocial behavior toward both close others and strangers [39]. We used the version of the CLS that has “humanity/strangers” as the target (e.g., *If a stranger is troubled*, *I usually feel extreme tenderness and caring*).

Finally, participants completed the Implicit Person Theories (IPT) scale, a measure of Dweck et al.’s entity versus incremental theories [40]. Participants responded to each item on a 7-point scale with endpoints labeled *Strongly Disagree* (1) and *Strongly Agree* (7). After reverse coding such that high scores reflected an entity theory (e.g., *Everyone is a certain kind of person*, *and there is not much they can really do to change that*), the items were averaged.

### Results and discussion

Table 1 provides means, standard deviations, Cronbach’s alpha, and correlations among all measures. We computed a path model to test our mediational hypothesis. Unless otherwise noted, all mediational path models were computed using Hayes’s PROCESS program for SPSS [41]. Also, prior to computation of all path models in this article, variables were standardized so that path diagrams reflect standardized solutions. In the present study, we used Hayes’s Model 4.

**Table 1:** Descriptive statistics and correlations among variables (Study 1).

	*M* [possible range]	*SD*	*α*	1.	2.	3.	4.	5.
1. Lay Historicism	4.06 [1–5]	.62	.83					
2. Lay Controllability Theory	4.13 [1–5]	.72	.87	.06				
3. Perceived Suffering	3.98 [1–5]	.61	.82	.41***	-.04			
4. Compassion-proneness	4.51 [1–7]	.91	.94	.21*	.09	.24**		
5. Implicit Person Theories	3.73 [1–7]	1.16	.88	-.04	-.08	.06	-.13	
6. Sex [0 = Male, 1 = Female]	.55	.50	n/a	.09	-.02	.02	.19*	.06

Note.

*p < .05

**p < .01

***p < .001.

**Fig 1:**
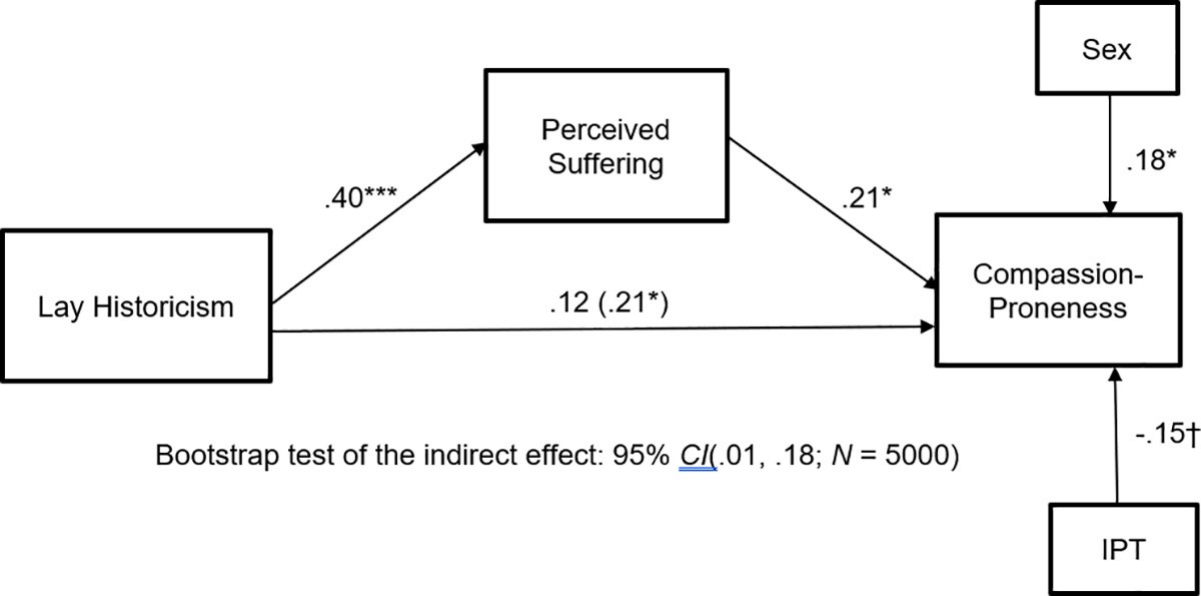
Study 1: Lay historicism contributes to dispositional compassion proneness via increased perceptions of suffering. Coefficients are standardized. Sex is coded such that 0 = Male, 1 = Female. †*p* = .06, *p < .05, ***p < .001.

Fig 1 depicts the model that we tested, which controlled for the effects of sex and implicit person theories on compassion proneness. As can be seen by looking at Table 1, removing the covariates has virtually no impact on the associations tested. As can be seen from the model coefficients in Fig 1, lay historicism was positively associated with the perception that past suffering lies behind current bad actions and choices, *t*(147) = 5.52, *p* < .001, and this perception was, in turn, positively related to compassion proneness, *t*(146) = 2.36, *p* = .019. This was a significant indirect effect as indicated by the bootstrapping results presented beneath the model. These results occurred while controlling for the marginal link between IPT and compassion proneness, *t*(146) = -1.87, *p* = .06, and the significant link between biological sex and compassion proneness, *t*(146) = 2.33, *p* = .021.

These results conceptually replicate earlier findings in which historicist narratives elicited compassion for an outgroup by creating a sense that the outgroup had suffered [15]. The present results go beyond that work, however, by showing that the link between historicism and compassion—mediated by perceived suffering—operates at the level of abstract lay theories—lay historicism—and broad dispositions—general compassion proneness—and not only at the level of a particular beliefs about a particular group whose history is explicitly provided.

## Study 2: The unique contribution of lay historicism to compassion proneness

The purpose of Study 2 is to examine whether lay historicism provides unique information about compassion proneness, above and beyond the information provided by other well-known contributors to compassion.

### Method

#### Participants

Participants were 168 individuals (69 male) who participated via Amazon’s Mechanical Turk interface. Their average age was 36 years (*SD* = 13).

#### Procedure

After providing informed consent, participants completed our measures of *lay historicism* and *lay controllability theories*. As an alternative measure of compassion proneness, they completed the *empathic concern* subscale of Davis’s Interpersonal Reactivity Index (IRI; e.g., *I would describe myself as a pretty soft-hearted person*; [2]).

Participants also completed measures of several additional well-established predictors of compassion. One such measure was the perspective taking subscale of Davis’s IRI [e.g., *When I’m upset at someone*, *I usually try to “put myself in his shoes” for a while*; 2]. Another measure was McFarland, Webb, and Brown’s Identification with All Humanity (IWAH) scale [4]. This measure taps identification with “people in my community,” “Americans,”, and “people all over the world” (e.g., *How close do you feel to… People in my community*? *Americans*? *People all over the world*?). McFarland et al. showed that human identification—rather than community or American identification—was especially predictive of a broad tendency toward compassion. They also noted the importance of separating the effects of human identity from the tendency to identify with the more specific groups. Toward that end, following procedures in [4], we regressed human identity on both community identification and American identification and saved the residuals to use as our indicator of identification with all humanity. Finally, participants completed the IPT scale [39] as in Study 1 and some demographic items that included biological sex.

### Results and discussion

Table 2 provides means, standard deviations, Cronbach’s alpha, and correlations among all measures. As can be seen there, all predictor variables were significantly related to empathic concern in the expected direction. The relation between perspective taking and empathic concern was very large, possibly because items for those scales were crafted to be subcomponents of a single empathy scale. This exceedingly strong relation enables us to conduct a stringent test of whether lay historicism contributes to compassion proneness beyond other well-known predictors. Once again, lay historicism was unrelated to entity/incremental theories.

**Table 2:** Descriptive statistics and correlations among variables (Study 2).

	*M* [possible range]	*SD*	*α*	1.	2.	3.	4.	5.	6.
1. Lay Historicism	3.82 [1–5]	.68	.87						
2. Lay Controllability Theory	4.17 [1–5]	.76	.89	.24**					
3. Perspective Taking	3.77 [1–5]	.76	.90	.22**	.26**				
4. IWAH	3.02 [1–7]	.79	.90	.25**	.13	.17*			
5. Implicit Person Theories	3.52 [1–7]	1.34	.95	.06	-.09	-.15†	-.21**		
6. Sex [0 = M, 1 = F]	.59	.49	n/a	.19*	.06	.26**	.04	-.04	
7. Empathic Concern	3.71 [1–5]	.77	.90	.31***	.18*	.62***	.34***	-.19*	.33***

*Note*. For IWAH (Identification with All Humanity), descriptive statistics are for the raw scale score. Correlations are based on the residualized measure described in the body of our article.

†*p* = .06

*p < .05

**p < .01

***p < .001.

We computed a multiple regression analysis to determine whether lay historicism is a unique contributor to compassion proneness. Results are in Table 3. As can be seen there, all predictors except lay controllability theories and IPT contributed significantly and independently to the prediction of compassion proneness. The sex effect indicates that women are more compassion-prone than men.

**Table 3:** Multiple regression predicting empathic concern (Study 2).

	β	*t*(161)	*p*
Lay Historicism	.13*	**2.12**	**.036**
Lay Controllability Theory	-.03	-.44	.662
Perspective Taking	.51***	**8.31**	**< .001**
IWAH	.20**	**3.29**	**.001**
IPT	-.07	-1.25	.215
Participant Sex	.17**	**2.79**	**.006**

*Note*. For participant sex, 0 = male, 1 = female. Significant effects are bolded. *IWAH* = Identification with All Humanity; *IPT* = Implicit Person Theories.

These analyses suggest that lay historicism provides novel information about compassion proneness. Although this is important evidence, we must also highlight its limitations. Indeed, the logic of emphasizing “unique variance accounted for” by each predictor side-steps the theoretically substantive issue of the interrelations among these constructs. Although lay historicism predicts compassion proneness beyond the effects of perspective taking and IWAH, it is also the case that historicism is positively related to both of these other constructs and that the effect of historicism was reduced in size when these other variables were added to the regression model. What does this mean? There are many possibilities. Perhaps those who are prone to perspective taking are relatively likely to become lay historicists due to their habit of looking deeply into others’ experiences. Furthermore, those with strong human identity are known to be motivated to learn about the experiences of others [4], which should contribute to the development of a historicist perspective on others. Thus, although the variables examined in this study each contribute independently to compassion proneness, we urge future research to focus on how these variables influence each other’s development and functioning. For now, though, it is important to know that lay historicism is not simply redundant with other well-known predictors.

## Study 3: Lay historicism fosters compassion for a specific individual via imputation of an unfortunate history

In Studies 1 and 2, compassion was examined as a general disposition. Study 3 will instead examine compassion for a specific individual. Specifically, we will test whether the mediational mechanism identified in Study 1—*Lay Historicism* → *Perceived Suffering* → *Compassion*—operates at the level of responses to a particular target person.

We hypothesized that this mechanism is likely to be operative when no history information about the target individual is explicitly provided. In such cases, the lay historicist theory will be used to “fill in the blank” about the target’s life history. That is, lay historicists *impute* the existence of an unfortunate life history. In contrast, when information about an unfortunate life history is explicitly presented, both lay historicists and non-historicists will perceive a history of suffering (Because they were directly told about it). This direct, equivalent perception of a history of suffering by both lay historicists and non-historicists should nullify the first link in our mediational model (i.e., *Lay Historicism* → *Perceived Suffering*).

### Method

#### Participants

Sixty-seven participants (44 female) participated for credit in their introductory psychology course.

#### Procedure

At a pretest, participants completed our measures of *lay historicism* and *lay controllability theory*. Several weeks later, they reported individually to the lab. There, they read two different vignettes regarding a college student who behaved in an antisocial manner. The first vignette described “David” who “appears to put little effort into making friends…like he just doesn’t like anyone… His apathetic attitude leaves him friendless” (see the S1 File for full text). The first vignette represented the *no history condition*, in which participants learned only of David’s disagreeable behavior and nothing of his life history. The second vignette began by noting that it would be highly similar—but not identical—to the first. The second vignette described “Michael,” whose behavior was described in precisely the same way as David’s behavior. But, for Michael a historicist narrative was presented: “[His behavior] has a lot to do with growing up in a cold and critical household. … [which gave him] a deep sense of being a generally unlikable person” (see S1 File for full text). One limitation of this study is that the order of vignettes was not counterbalanced. We note, however, that we find it difficult to imagine how this particular order of vignettes would create our predicted interaction for artifactual reasons.

Following each vignette, participants completed two items tapping *compassion* (e.g., *I feel compassion for David [Michael]*) and one item tapping *perceived suffering* (*David [Michael] has experienced a lot of pain in his life*).

### Results and discussion

Means, standard deviations, Cronbach’s alpha (or Pearson’s *r* for two-item scales), and correlations among our variables are presented in Table 4. Our key prediction was that lay historicism would be positively associated with perceptions of suffering in the no history condition, but not in the historicist narrative condition.

**Table 4:** Descriptives and correlations among variables (Study 3).

	*M* [possible range]	*SD*	*α* [or *r* for 2-item scales]	1.	2.	3.	4.	5.
1. Lay Historicism	4.01 [1–5]	.66	.85					
2. Lay Controllability Theory	4.58 [1–5]	.51	.86	-.12				
3. Perceived Suffering—No History Condition	3.33 [1–5]	.85	n/a [1 item]	.30*	-.28*			
4. Perceived Suffering—Historicist Narrative	4.61 [1–5]	.58	n/a [1 item]	.12	.10	.49***		
5. Compassion—No History Condition	3.19 [1–7]	.94	.79	.32**	-.12	.40**	.50***	
6. Compassion—Historicist Narrative	4.37 [1–5]	.74	.77	.33**	.08	.40**	.66***	.60***

*Note*. No History versus Historicist Narrative was a within-participants manipulation.

*p < .05

**p < .01

***p < .001.

**Fig 2:**
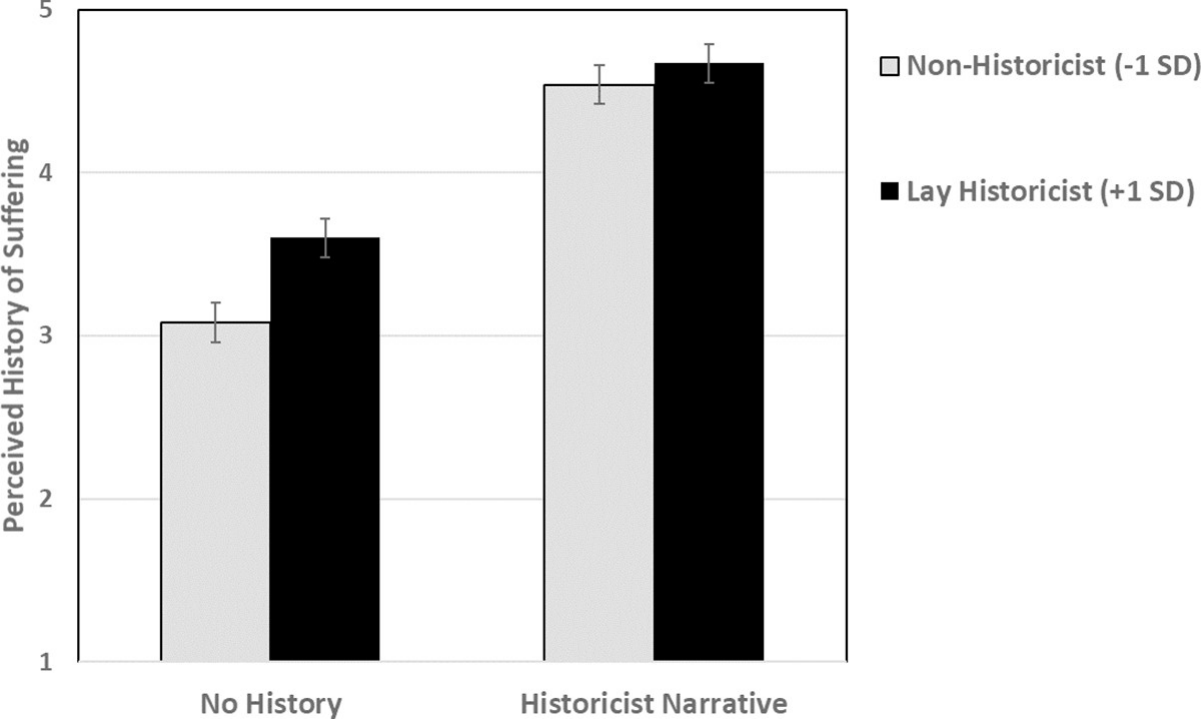
Study 3: Lay historicists impute a history of suffering when no such information is explicitly provided (no history condition), but do not differ from non-historicists in perceptions of suffering when such information is explicitly provided (historicist narrative condition). Errors bars are +/- SEM.

We tested this prediction using a repeated-measures GLM examining perceived suffering as a function of condition (within-participants: no history, historicist narrative), lay historicism (centered), and their interaction. This revealed a main effect of lay historicism, *F*(1, 64) = 4.70, *p* = .03 (*η*^2^ = .07) and a main effect of condition, *F*(1, 64) = 196.64, *p* < .001 (*η*^2^ = .75; see Table 4 for means). As predicted, these main effects were qualified by a condition X lay historicism interaction, *F*(1, 64) = 4.22, *p* = .04 (*η*^2^ = .06). The pattern of the interaction is shown in Fig 2. As can be seen there, lay historicism was associated with heightened perceptions (imputation) of suffering in the no history condition (where no such history was explicitly presented), *t*(64) = 2.53, *p* = .01 (*β* = .30). In contrast, in the historicist narrative condition, where information about a history of suffering was explicitly presented, lay historicism was unrelated to perceptions of suffering, *t* < 1 (*β* = .12). Both historicists and non-historicists, as seen in Fig 2, perceive suffering to an equal degree when an unfortunate history is explicitly presented.

**Fig 3:**
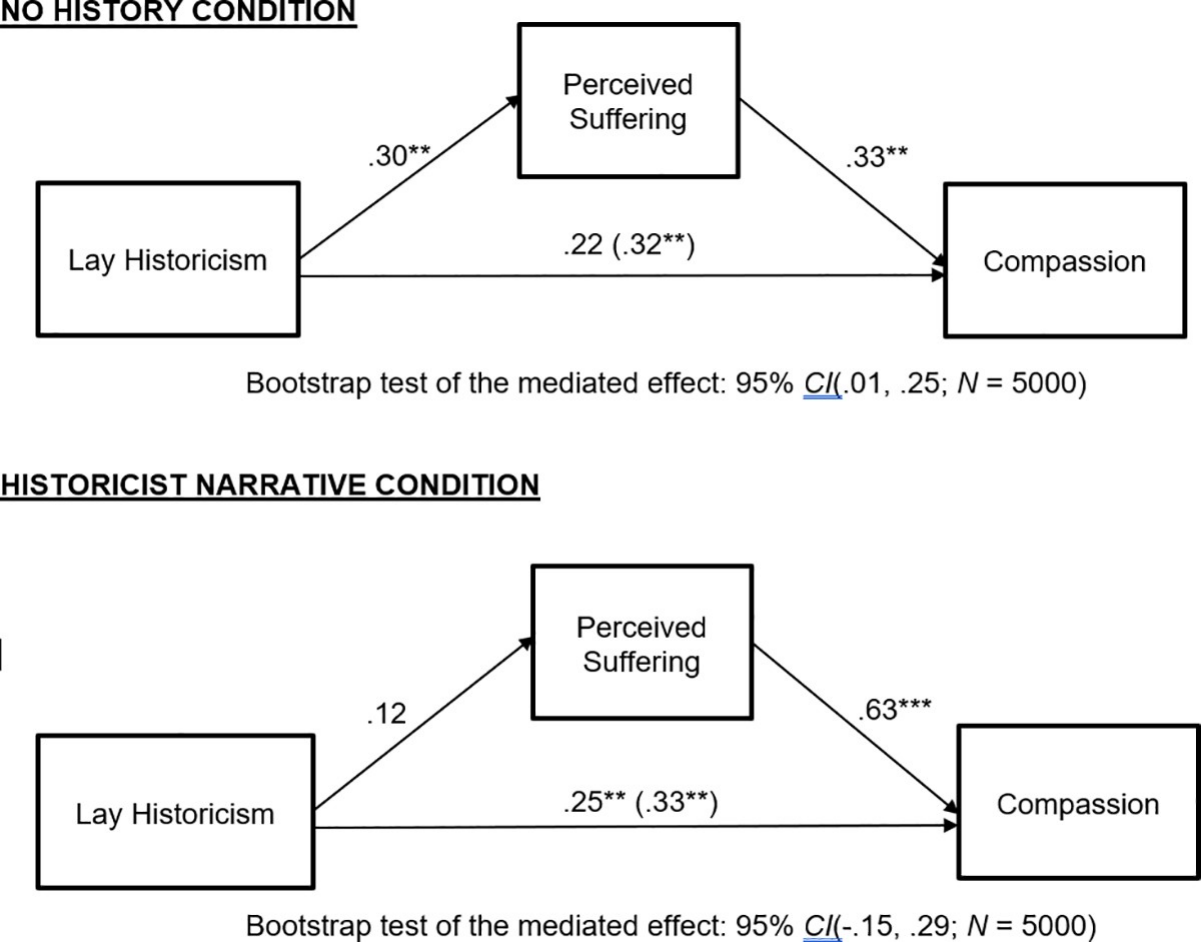
Study 3: The *Historicism → Perceived Suffering → Compassion* mediation model holds in the no history condition but not in the historicist narrative condition. Coefficients are standardized. *p < .05, **p < .01, ***p < .001.

This interaction pattern suggests that the mediational model explored above—*lay historicism* → *perceived suffering* → *compassion*—should hold in the present study within the no history condition but not within the historicist narrative condition. To explore this prediction, we computed the mediational path model separately within each condition (to our knowledge, there is no established technique for testing moderated mediation in a within-participants design). Results can be seen in Fig 3. As can be seen there, there was a significant indirect effect traveling from lay historicism to compassion through perceived suffering within the no history condition but not within the historicist narrative condition. This provides information about how the lay theory operates: It leads the historicist to infer the presence of an unfortunate history—and suffering—even when such information is not explicitly presented. We note here that the strong relation between perceived suffering and compassion in the historicist narrative condition argues against the possibility that the non-relation between lay historicism and perceived suffering in that condition is due to a ceiling effect involving perceived suffering.

## Study 4: Lay historicism, control of self-formation, and mitigation of harsh blame

Studies 1, 2, and 3 focused on the link between lay historicism and compassion. Study 4 will extend our focus by examining the link between lay historicism and the mitigation of blame. Although one might imagine that compassion and blame are the inverse of one another, there is clear evidence that they are sometimes completely dissociated [12]. This suggests that the tendency for lay historicism to increase compassion via perceived suffering might have little implication for whether (and why) lay historicism fosters mitigation of blame. Thus, here we seek evidence regarding the link between lay historicism and harsh blame. Our key hypothesis—informed by [12]—is that lay historicism will be associated with reduced belief in control of self-formation which, in turn, will predict reduced tendency for harsh blame responses.

### Method

#### Participants

Participants were 172 individuals (57 male) who participated via Amazon’s Mechanical Turk interface. Their average age was 41 years (*SD* = 15).

#### Procedure

After providing informed consent, participants completed an unrelated experiment. Next, they answered a variety of demographic questions. Then, they completed our measures of *lay historicism* and *lay controllability theory*.

Drawing heavily from the conceptualization and measures in prior work [12, 16, 17], we created a measure of general belief in control of self-formation (e.g., *People forge their own character*, *individually controlling the type of person they become over time*) and a measure of general belief in freedom of action (e.g., *Human beings have free will*: *The ability to choose*, *in every moment*, *how they will behave*). Following appropriate reverse-scoring, each participant received a score for *control of self-formation* beliefs and for *freedom of action* beliefs by computing the average across the scale items.

To provide validity evidence for these two scales, we ran a study (*N* = 109, recruited via MTURK) in which participants read about the office bully described in [12]. Next, participants rated the extent to which he possessed control of self-formation and freedom of action with respect to his bullying. These items were same as those used in [12]. Next, participants completed many filler items, including the Big Five Inventory and a couple pages of demographic items. Finally, they completed the general belief in control of self-formation and general belief in freedom of action scales described in the preceding paragraph. We regressed ratings of the office bully’s perceived control of self-formation on the general belief in control of self-formation and general belief in freedom of action scales. This revealed a significant effect of general belief in control of self-formation, *t*(106) = 3.96, *p* < .001 (β = .47) and no effect of general belief in freedom of action, *t*(106) = .99, *p* = .32 (β = .12). Next, we regressed ratings of the office bully’s perceived freedom of action on the general belief in control of self-formation and general belief in freedom of action scales. This revealed a significant effect of general belief in freedom of action, *t*(106) = 3.14, *p* = .002 (β = .40) and no effect of general belief in control of self-formation, *t*(106) = .37, *p* = .71 (β = .05). These results provide further evidence for Gill and Cerce’s claim that everyday people distinguish between freedom of action and control of self-formation as distinct concepts of free will, and further suggests that our general belief scales successfully discriminate between those two concepts.

Finally, participants completed the Blame Intensity Inventory (BII) [42]. The BII comprises seven items designed to assess the respondent’s propensity for harsh, punitive blame responses. Each item describes a person who behaved badly: e.g., *You learn about a Wall Street investor who lied to many people in order to steal their money*. *He used the money to fund a lavish lifestyle*. Next, there is a description of a harsh, punitive blame response: e.g., *What is the likelihood that you would strongly blame him*, *detesting him and hoping that he spends many miserable years behind bars*? The items were averaged to form an index of *harsh blame*. Harsh blame is particularly important as it can lead the blamer to take pleasure in the suffering of the transgressor [42], the antithesis of a humane response.

Beyond testing whether lay historicism mitigates harsh blame, we sought to provide evidence that this effect was independent of the effect of other broad cognitive style variables. Accordingly, we included a measure of Need for Cognitive Closure (NFCC) [43]. NFCC has been linked to a preference for hostile approaches to conflict situations [44].

### Results and discussion

Table 5 presents means, standard deviations, Cronbach’s alpha, and correlations among all measured variables. Interestingly, although general belief in control of self-formation and general belief in freedom of action were both positively related to lay controllability theories (as one should expect), a *z*-test for dependent correlations suggested that the relationship was significantly stronger for belief in freedom of action, *z* = 2.48, *p* = .007. This fits with an implication of prior work that freedom of action might be the more accessible lay concept of free will [12], and thus more likely to come immediately to mind if one is asked whether a person “has control” (as the question is posed on the SESQ). Control of self-formation is a somewhat more abstract concept, as it involves thinking about how personality is formed over an extended period.

**Table 5:** Descriptive statistics and correlations among variables (Study 4).

	*M* [possible range]	*SD*	*α*	1.	2.	3.	4.	5.
1. Lay Historicism	3.77 [1–5]	.67	.87					
2. Lay Controllability Theory	4.42 [1–5]	.67	.91	-.04				
3. Control of Self-Formation	4.30 [1–7]	1.17	.95	-.17*	.34***			
4. Freedom of Action	4.93 [1–7]	.93	.88	-.16*	.48***	.66***		
5. Harsh Blame	5.14 [1–7]	.91	.75	.07	.25**	.31***	.28***	
6. NFCC	3.89 [1–6]	.59	.90	.04	.22**	.30***	.35***	.42***

*Note*.

*p < .05

**p < .01

***p < .001. *NFCC* = Need for Cognitive Closure.

**Fig 4:**
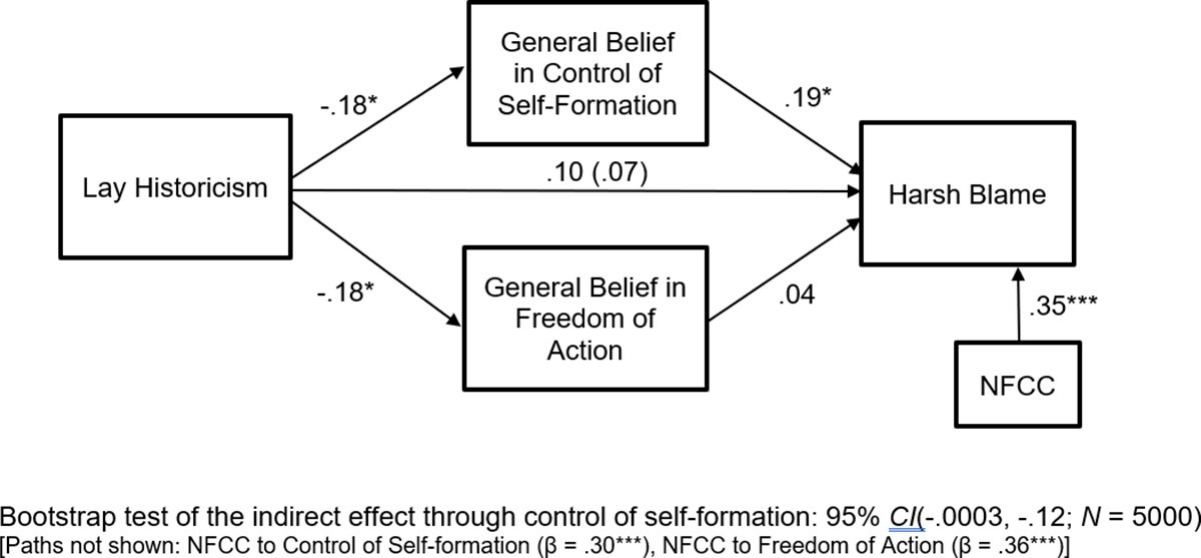
Study 4: Lay historicism tempers blame responses via diminished belief in control of self-formation. Coefficients are standardized. *p < .05, ***p < .001.

We computed the mediational path model shown in Fig 4. As can be seen from the model coefficients there, the results revealed support for a blame-mitigating effect of lay historicism. Specifically, lay historicism was associated with reduced belief in both control of self-formation, *t*(169) = -2.45, *p* = .015, and freedom of action, *t*(169) = -2.48, *p* = .014. Furthermore, belief in control of self-formation was positively related to harsh blame, *t*(167) = 2.11, *p* = .036, although belief in freedom of action was not, *t*(167) = .47, *p* = .64. Taken together, these relations suggest that lay historicism mitigates harsh blame by reducing the general belief that transgressors are the architects of their own moral character. This indirect effect was statistically significant according to the bootstrapping results shown at the bottom of Fig 4.

Using Hayes’ PROCESS program [41], we computed a bootstrap test (*N* = 5000) of the difference between the indirect effects traveling through control of self-formation and freedom of action. This revealed that the 95% confidence interval for the difference between indirect effects included zero (-.04, .12), as did the 90% confidence interval (-.03, .10). Thus, although the indirect effect traveling through control of self-formation was significant and the indirect effect traveling through freedom of action was not, the two indirect effects were not significantly different from each other. Importantly, Study 5 will conceptually replicate the present study to see if the relative superiority of control of self-formation as a mediator replicates. All these effects obtained while controlling for the positive relation of NFCC to control of self-formation, *t*(169) = 4.21, *p* < .001, to freedom of action, *t*(169) = 5.09, *p* < .001, and to harsh blame, *t*(167) = 4.72, *p* < .001.

The mediated effect traveling through diminished perceptions of control of self-formation conceptually replicates prior experimental findings involving historicism [12]. This is important because it shows that lay historicists have a general expectation that individuals lack full control of self-formation, and control of self-formation has been shown in prior work to play a critical role in blame mitigation in individual cases. The results go beyond prior work, however, by suggesting that the link between historicist thinking and the mitigation of harsh blame operates at the level of general, abstract beliefs about the social world and not only in the case where concrete historical information is explicitly provided about a particular offender.

One difference from prior work is that here we found a link between lay historicism and diminished belief in freedom of action. In contrast, Gill and Cerce found that an experimental manipulation of historicist narratives (present, absent) had no effect on perceived freedom of action [12]. Why might this be? One possible explanation involves the types of transgressions studied by Gill and Cerce: Highly intentional transgressions. Because they involve a high level of apparent choice, highly intentional transgressions are seen as necessarily involving high levels of freedom of action [45]. In contrast, the SESQ measures lay historicism using at least some scenarios that could be construed as a bit more impulsive (e.g., being argumentative, being adulterous, losing one’s temper). When a person’s history explains how she developed an impulsive habit, then history might be seen as reducing freedom of action. Thus, plausibly, historicist thinking can reduce both perceived control of self-formation and perceived freedom of action depending on which types of actions are being considered. Future research is needed to explore these links more carefully. Finally, although lay historicism predicted decreased belief in freedom of action in the present study, the mediational results suggesting a potentially more important mediating role for control of self-formation is consistent with prior work [12]. Interestingly, control of self-formation also mediated the effect of lay controllability theories on harsh blame (see S2 File).

## Study 5: Lay historicism specifically regarding transgressors

Study 1 showed that lay historicism was associated with heightened compassion proneness, mediated via perceived suffering. Study 4 showed that lay historicism was associated with diminished tendency toward harsh blaming, mediated via reductions in perceived control of self-formation. Study 5 will include both mediators to test whether lay historicism predicts blame mitigation *only* via reductions in perceived control of self-formation and *not* via perceived suffering. If so, this would establish a noteworthy parallel: Mediation of the effect of lay historicist *theories* on blame mitigation happens in the same way as mediation of case-specific manipulation of historicist narratives (i.e., via control of self-formation and not via perceived suffering; [12]).

Related to this point about distinct mediators, we collected additional data from a sample of MTURK Workers (*N* = 225). They completed the general belief in control of self-formation scale from Study 4 and the Compassionate Love Scale from Study 1. There was no correlation between the two scales, *r*(223) = .04, *p* = .52. In a second study using college undergraduates (*N* = 72), we found a similar lack of relation between general belief in control of self-formation and the Empathic Concern scale from Study 2, *r*(70) = -.003, *p* = .98. These results cast doubt on a link between control of self-formation and compassion, and thus suggest that control of self-formation is unlikely to mediate the link between lay historicism and compassion. Thus, we have some evidence for distinct mediation of the link between lay historicism and compassion via perceived suffering.

Beyond these issues regarding distinct mediation, in Study 4, the link between lay historicism and diminished perceptions of free will was small. We wondered if we might find larger effect sizes if we made our measure of lay historicism a bit narrower in scope. Accordingly, we developed a measure of lay historicist beliefs specific to the development of immoral or criminal character. Indeed, existing evidence suggests that negative actions lead people to think more carefully about issues of choice and control [46]. Such deeper processing could lead to measures of lay historicism regarding bad character relating more strongly to measures of free will than do broader measures such as the SESQ. We note that there is ample precedent for varying the level of abstractness at which lay theories are measured [40, 47–50].

### Method

#### Participants

Participants were 202 individuals (102 male) who participated via Amazon’s Mechanical Turk interface. We used a memory check procedure in which participants needed to identify which topics were covered in the surveys they completed. Only participants who scored 100% were included in our final sample. This reduced our sample size to 188 participants (93 male) with an average age of 34 years (*SD* = 12).

#### Procedure

After providing informed consent, participants completed a measure of *lay historicism* regarding the development of moral character (e.g., *A person who behaves badly became that way due to an unfortunate or difficult life history*; *Life history does NOT have a very powerful effect on moral character development*). After appropriate reverse scoring, the 11 items were averaged to form an index of lay historicism regarding transgressors. Because we did not use the SESQ, the lay controllability theories subscale was not collected here. Control of self-formation and freedom of action were each assessed using three items. The three items were taken directly from prior work [12], but with the target’s name (e.g., *James*) replaced with the generic phrases of “a bad person” or “an immoral person” (e.g., control of self-formation: *Throughout his or her life*, *a bad person is always in control of his/her personality development*; freedom of action: *It is possible for an immoral person to choose to behave differently*). The three items composing each scale were averaged to form indexes of *control of self-formation* and *freedom of action*. Perceived suffering was measured using four items that were modeled after prior work [31] but replaced specific group labels with more generic labels referring to transgressors (e.g., *Most criminals have previously experienced a lot of emotional suffering and frustration*). After appropriate reverse scoring, the items were averaged to form an index of *perceived suffering*. Finally, participants completed nine items to tap harsh blame reactions. In contrast to the BII scale used in Study 4, these were written as generalizations (e.g., *A bad person should be made to suffer for his/her bad deeds*), rather than as responses to concrete transgressions. The items were averaged to form an index of *harsh blame*.

### Results and discussion

Means, standard deviations, Cronbach’s alpha, and correlations among variables are presented in Table 6. As can be seen there, our more specific measure of lay historicism was associated with reduced belief in both control of self-formation and freedom of action, and with increased perceptions of suffering. Here, however, the negative relation of lay historicism with control of self-formation was roughly twice as large as in Study 4, and more than twice as large as its relation with freedom of action in the present study. According to a *z*-test for dependent correlations, the link between lay historicism and control of self-formation was significantly larger than the link between lay historicism and freedom of action, *z* = 2.99, *p* = .003 (two-tailed).

**Table 6:** Descriptives and correlations among variables (Study 5).

	*M* [possible range]	*SD*	*α*	1.	2.	3.	4.
1. Lay Historicism	3.49 [1–5]	.66	.89				
2. Control of Self-Formation	3.38 [1–5]	.90	.84	-.34***			
3. Freedom of Action	4.00 [1–5]	.69	.75	-.15*	.59***		
4. Perceived Suffering	2.98 [1–4]	.51	.77	.60***	-.21**	-.06	
5. Harsh Blame	4.87 [1–7]	1.03	.89	-.04	.27***	.22**	.05

*Note*.

*p < .05

**p < .01

***p < .001.

**Fig 5:**
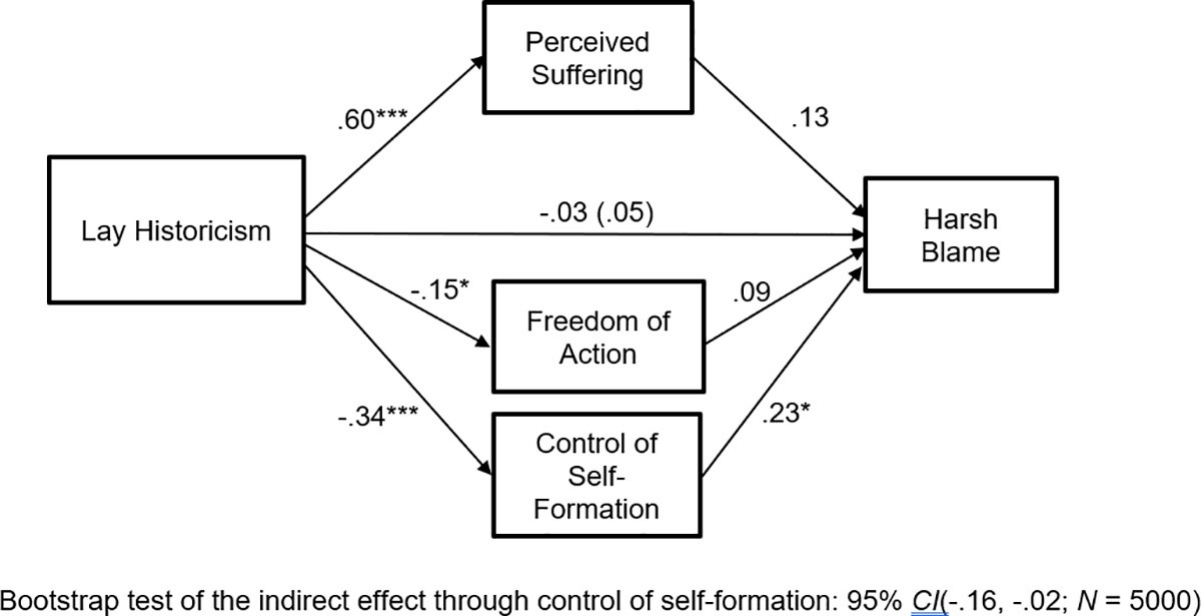
Study 5: Lay historicism specifically regarding transgressors—a narrower measure than the SESQ—reduces harsh blame via diminished belief in control of self-formation. Coefficients are standardized. *p < .05, ***p < .001.

Next, we computed the mediational path model shown in Fig 5. As can be seen from the model coefficients there, the strongest link in the model was a positive association between lay historicism and perceptions of suffering, *t*(185) = 10.21, *p* < .001. Yet, perceived suffering was unrelated to blame, *t*(182) = 1.44, *p* = .15. This perfectly replicates the patterns identified by previous experimental studies of historicist narratives [12]. Lay historicism was also associated with moderate reductions in perceived control of self-formation, *t*(185) = -4.91, *p* < .001, and small reductions in perceived freedom of action, *t*(185) = -1.99, *p* = .048. Furthermore, replicating Study 4 (and [12]), only control of self-formation carried an effect of lay historicism blame mitigation, *t*(182) = 2.56, *p* = .011. Replicating Study 4, freedom of action was unrelated to harsh blame, *t*(182) = .99, *p* = .32. As can be seen at the bottom of the figure, the indirect effect traveling through control of self-formation was statistically significant according to a bootstrap test.

Using Hayes’ PROCESS program [41], we computed a bootstrap test (*N* = 5000) of the difference between the indirect effects traveling through control of self-formation and freedom of action. This revealed that the 95% confidence interval for the difference between indirect effects included zero (albeit barely: -.01, .16), whereas the 90% confidence interval did not include zero (.001, .14). This is akin to finding that the difference was marginal in a two-tailed test but significant in a one-tailed test.

This study replicates the *Lay Historicism* → *(reduced) Belief in Control of Self-Formation* → *Blame* mechanism shown in Study 4 and provides evidence for a stronger link between lay historicism and diminished perceptions of control of self-formation than was found there. One key to finding this stronger effect appears to be our utilization of a slightly more specific measure of lay historicism: A measure focused on the development of bad moral character. Related to this point, we reanalyzed all the studies in this paper using the four negative behavior items from the SESQ and the four positive behavior items from the SESQ as separate predictors (see S2 File). Overall, the results are not entirely consistent. But, there was at least a bit of evidence suggesting that historicism regarding negative behaviors was a better predictor of some outcomes than was historicism regarding positive behaviors. Future research should explore this more thoroughly using more clearly separate measures of positive and negative historicism.

## Study 6: Lay historicism and criminal justice attitudes

In Study 6, we leave behind the questions of mediation that occupied us in Studies 1–5 and shift to a focus on the relation between lay historicism and indices humane responding that are more grounded in particular real-world issues. That is, in Study 6, we will measure people’s endorsement of several different criminal justice philosophies: Harshness, rehabilitation, quarantine, and prevention.

### Method

#### Participants

Five-hundred fifty-eight participants (293 female) participated for credit in their introductory psychology course. Their average age was 19 (*SD* = 1).

#### Procedure

After providing informed consent, participants completed our measure of *lay historicism* and *lay controllability theory* from the SESQ. They also completed a measure of *Criminal Justice Philosophies* that was created based on the conceptual dimensions and items developed by many criminal justice scholars [51]. Four items tapped *harshness*, with items including support for the death penalty and general endorsement of harshness (*I think the criminal justice system needs to deal more harshly with people convicted of crimes*). Next, five items tapped support for *rehabilitation* (*Prisoners should have access to psychological services—e*.*g*., *counseling—while in prison*, *to help change the patterns of emotion and behavior that contribute to their illegal acts*). Two items tapped support for *quarantine*, or an emphasis of keeping criminals “off the streets” with no explicit reference to how they will be treated in prison (*A crucial function of prisons is to protect society*: *Dangerous criminals must be kept behind bars*). Finally, three items tapped support for *prevention*, such as early intervention programs focused on preventing crime by improving communities (*A major priority should be to invest in ways to prevent kids from taking wrong turns and getting tangled up in gangs*, *violence*, *or prison*). The existence of these four related-yet-distinct criminal justice philosophies is well-established in the literature [51].

Because political ideology is a well-established predictor of criminal justice philosophies [51], we included a single-item measure of ideology that ranged from 1 (*Very Liberal*) to 7 (*Very Conservative*). This enables us to test whether lay historicism predicts criminal justice philosophies beyond effects of ideology.

### Results and discussion

Means, standard deviations, Cronbach’s alpha, and correlations among our variables are presented in Table 7. Endorsement of each criminal justice philosophy was significantly different from endorsement of each other philosophy. Harshness was the least popular, endorsed significantly less than all the others [*t*s(553) = 9.88, 18.20, 19.55, all *p*s < .001, for rehabilitation, quarantine, and prevention]. Rehabilitation was endorsed significantly less than both quarantine and prevention [*t*s(553) = 3.98, 14.41, *p*s < .001, respectively]. Prevention was the most strongly endorsed philosophy [*t*(553) = 7.00, p < .001, versus quarantine].

**Table 7:** Descriptives and correlations among variables (Study 6).

	*M* [possible range]	*SD*	*α*	1.	2.	3.	4.	5.	6.
1. Lay Historicism	3.79 [1–5]	.62	.82						
2. Lay Controllability Theory	4.13 [1–5]	.67	.86	-.05					
3. Ideology	3.56 [1–7]	1.44	n/a [single item]	-.03	.01				
4. Harshness	4.26 [1–7]	1.49	.82	-.02	.10*	.31***			
5. Rehabilitation	5.11 [1–7]	1.28	.88	.16***	.01	-.22***	-.27***		
6. Quarantine	5.37 [1–7]	1.37	.73	.06	.19***	.15***	.39***	-.07	
7. Prevention	5.74 [1–7]	1.26	.92	.22***	.13**	-.16***	-.09*	.42***	.20***

*Note*.

*p < .05

**p < .01

***p < .001.

Our major interest was in whether lay historicism was associated with criminal justice philosophy preferences. Looking at Table 7, one can see that—compared to non-historicists—lay historicists were significantly more likely to endorse a rehabilitation philosophy and to support a philosophy of crime prevention via community investment. Table 8 presents regression analyses in which each criminal justice philosophy was regressed on lay historicism, lay controllability theories, and ideology. Even though ideology was significantly related to each criminal justice philosophy, and lay controllability theories were related to three of the four philosophies (i.e., all but rehabilitation), the relation of lay historicism to rehabilitative and preventive philosophies remained almost exactly the same even when ideology and lay controllability theories were statistically controlled. Thus, the effect of historicism is independent of the effects of these other variables.

**Table 8:** Multiple regression: Criminal justice philosophies as a function of lay theories and ideology (Study 6).

	Harshness		Rehabilitation		Quarantine		Prevention	
	*Β*	*t*(552)	*β*	*t*(552)	*β*	*t*(552)	*β*	*t*(552)
Lay Historicism	-.00	-.06	.16***	**3.76**	.07	1.71	.22***	**5.41**
Lay Controllability Theory	.10*	**2.38**	.02	.36	.20***	**4.70**	.15***	**3.50**
Ideology (high score = conservative)	.31***	**7.82**	-.21***	**-5.20**	.15***	**3.70**	-.15***	**-3.70**

*Note*. Significant or marginal effects are bolded.

*p < .05

***p < .001.

These patterns suggest that lay historicists are more supportive of humane alternatives in the criminal justice system than are non-historicists.

## Study 7: Lay historicism and blame of a peer in face-to-face interaction

Studies 1 through 6 assessed blame and compassion toward abstract social targets. In Study 7, participants had a face-to-face interaction with an actual peer. The peer was, in reality, a confederate who was working from a script. She described irresponsible behaviors that she had engaged in since arriving at college.

Our focus is on whether lay historicism would impact blame of this flesh-and-blood target person. Study 7 will also add a unique twist: In one condition, participants will learn that the irresponsible peer had a supportive formative history that *should have* (arguably) molded her into a responsible person. We expect that lay historicism might contribute to *exacerbation*—rather than mitigation—of blame in this case. If so, this would suggest that lay historicism is not simply about “being nice”; rather, it is about taking life history information into account when rendering judgments of others.

### Method

#### Participants

Eighty participants (41 female) participated for credit in their introductory psychology course.

#### Procedure

At a pretest, participants completed our measures of *lay historicism* and *lay controllability theory*. Roughly two months later, they reported individually to the lab. A female confederate arrived at the same time and pretended to be another participant from the introductory psychology pool. Both the participant and the confederate signed a consent form agreeing to participate in a study of “how people form first impressions.” Next, they were escorted to a new room and seated on opposite sides of a small table. The experimenter explained that the duo would engage in a “getting acquainted exercise” in which one would play the role of “interviewer” and the other would play the role of “interviewee.” The experimenter presented an envelope marked “roles” and asked the confederate to pull out a slip of paper. The envelope was rigged such that the confederate always got the role of interviewee.

Next, the experimenter turned to the real participant (the interviewer), presented a new envelope, and said, “Without looking, please select one [of these conversation starters] and read it to the interviewee.” For all participants, this first slip said, “Describe some difficulties you have experienced since starting college.” After the participant read this aloud, the confederate responded according to a prepared script. The script began as follows: *I guess I’ve had some difficulties being a good and responsible student*. Please see the S1 File for the full text.

Next, the interviewer was presented with a second envelope out of which he or she was to draw a second conversation topic. At this point, participants were randomly assigned to either the *no history* condition or the *supportive history* condition. In the no history condition, participants did not receive any historical information about the interviewee. Rather, the additional question was about the interviewee’s hobbies. The scripted reply (deliberately bland and uninformative) is available in the S1 File. In the supportive history condition, the interviewee’s response was designed to provide historical information that was relevant to the interviewee’s character development (i.e., “Tell me a little bit about your relationship with your parents.”). The scripted reply conveyed that the interviewee’s parents were highly supportive (see S1 File).

After the interview, the confederate and the participant were brought to separate rooms. The participant was given a survey packet containing questions about his or her impression of the interviewee. Some questions were answered on five-point scales [*Strongly Disagree* (1), *Strongly Agree* (5)] and others were answered on seven-point scales [*Not At All* (1), and *Very Much* (7)].

Participants first responded to the item, *My conversation partner has experienced some difficulties since starting college*. We included this item to ensure that the magnitude of the target’s dysfunction was viewed similarly by those low and high in lay historicism. If so, then any differences in blame likely reflect differences in blame *per se* rather than differences *perceived difficulties* (e.g., *Her difficulties are minor*, *so why would I blame her*?).

Next, we measured our primary dependent variable of *blame* with three items (e.g., *I think my conversation partner deserves most of the blame for her difficulties*). As an additional blame-related variable, we measured perceptions of the target’s *incompetence* (e.g., *lazy*, *unreliable*). The link between blame and attribution of negative character traits is well-established [52].

Finally, participants made additional judgments for which predictions were less clear-cut. These included items tapping *compassion* (e.g., *I felt sympathy for my conversation partner*), the target’s *pleasantness* during the conversation (e.g., *nice*, *pleasant*), *trait warmth* (e.g., *warm*, *likable*), and *identification* (e.g., *I am similar to my conversation partner*). We were not sure whether the impact of lay historicism on compassion would be moderated by our manipulation of history information. Because a person with a supportive history might be seen as suffering due to her inability to live up to her parents’ outstanding example, the lay historicist’s sensitivity to perceived suffering could lead to compassion in the supportive history condition.

### Results and discussion

Means, standard deviations, Cronbach’s alpha, and correlations among our variables are presented in Table 9. Our key prediction was that lay historicism would contribute to reduced blame of the target in the no history condition (cf. Study 3), but that the effect of lay historicism would be diminished or reversed in the supportive history condition. We expected that these predicted effects would show up in blame ratings and in derogatory ratings of incompetence.

**Table 9:** Descriptives and correlations among variables (Study 7).

	*M* [possible range]	*SD*	*α*	1.	2.	3.	4.	5.	6.	7.	8.
1. Lay Historicism	4.06 [1–5]	.64	.78								
2. Lay Controllability Theory	4.22 [1–5]	.65	.87	.08							
3. Magnitude of Struggles	3.74 [1–5]	.63	n/a [1 item]	.13	.09						
4. Blame	3.41 [1–5]	.72	.85	-.04	.04	.12					
5. Incompetence	3.69 [1–7]	1.06	.86	.04	-.12	.11	.49***				
6. Compassion	3.52 [1–5]	.68	.73	.23*	.06	.06	-.35**	-.27*			
7. Current Pleasantness	6.33 [1–7]	.57	.84	-.05	.17	.13	-.19	-.39***	.23*		
8. Trait Warmth	5.74 [1–7]	.73	.84	-.02	-.01	.13	-.16	-.39***	.25*	.69***	
9. Identification	3.19 [1–5]	.73	.66	.13	.01	.14	-.42***	-.56***	.48***	.33**	.30**

*Note*.

*p < .05

**p < .01

***p < .001.

We regressed each dependent variable on history condition (no history, supportive history), lay historicism (centered), and their interaction. Prior to testing our main predictions, we analyzed the single item concerning the magnitude of the target’s difficulties. Our regression analysis involving this variable revealed no significant effects, all *t*s(76) < 1. Thus, it is unlikely that the effects of lay historicism below can be explained in terms of differences in the perceived magnitude of the target’s difficulties.

**Fig 6:**
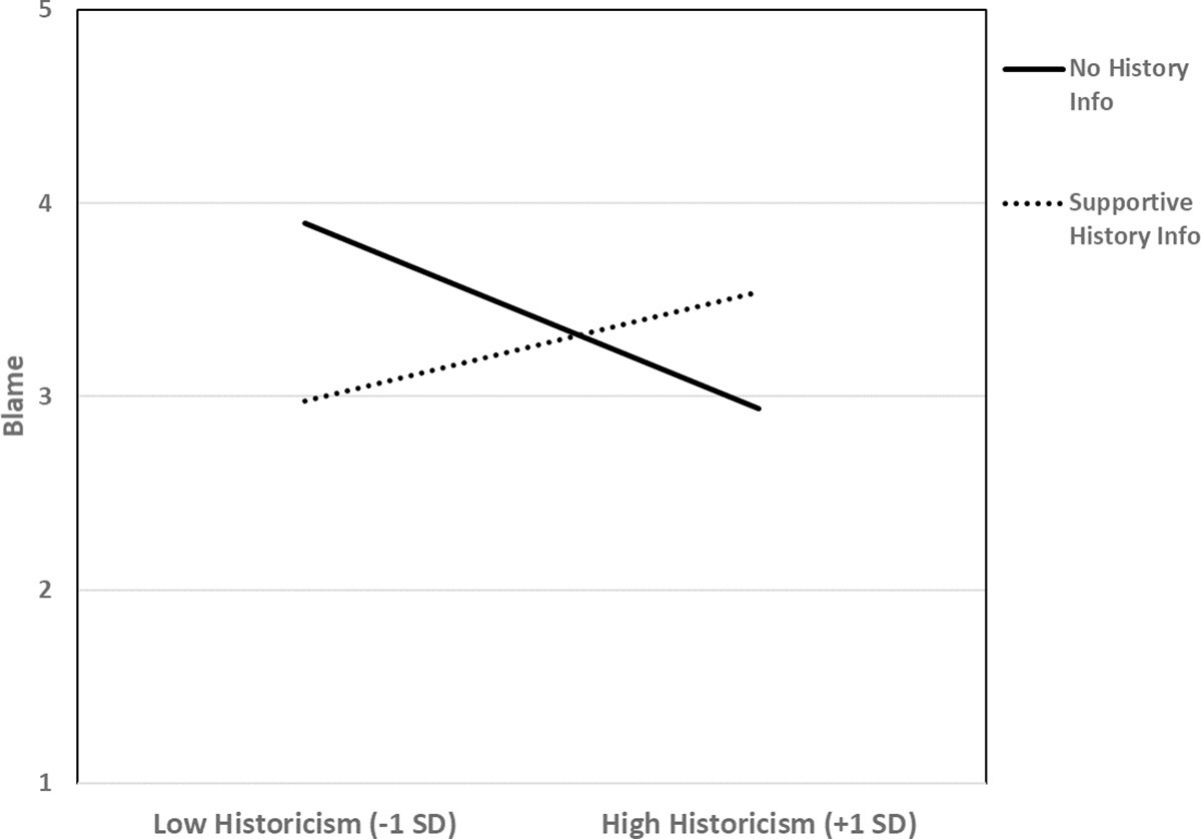
Study 7: Lay historicism is associated with blame mitigation when no life history information is given. When life history is described as supportive, however, lay historicism no longer predicts blame mitigation.

Next, we analyzed our remaining dependent variables using the regression model described above. Results are in Table 10. One primary dependent variable was blame. As can be seen in Table 10, our predicted lay historicism X history information interaction was significant. The pattern of the interaction can be seen in Fig 6. As can be seen there, as predicted, lay historicism was associated with reduced blame of the irresponsible peer in the no history condition, *r*(39) = -.37, *p* = .018. In contrast, there was a non-significant trend in the opposite direction in the supportive history condition, with lay historicists showing a trend toward blaming *more* than non-historicists, *r*(37) = .25, *p* = .12.

**Table 10:** Face-to-face interaction: Ratings of the confederate (Study 7).

	Blame		Incompetence		Compassion		Pleasant-ness		Trait Warmth		Identification	
	*β*	*t*(76)	*β*	*t*(76)	*β*	*t*(76)	*β*	*t*(76)	*β*	*t*(76)	*β*	*t(76)*
Lay Historicism (centered)	-.14	-1.23	-.06	-.55	**.22†**	**1.91**	-.03	-.21	.01	.05	.15	1.30
History Info (none, supportive)	-.03	-.32	-.01	-.07	.12	1.09	-.07	-.61	-.06	-.49	-.06	-.58
Lay Historicism X History Info	.34**	**2.98**	.35**	**3.08**	.02	.16	-.08	-.67	-.09	-.74	-.08	-.66

*Note*. Significant or marginal effects are bolded. †*p* = .06, *p < .05

**p < .01, ***p < .001.

**Fig 7:**
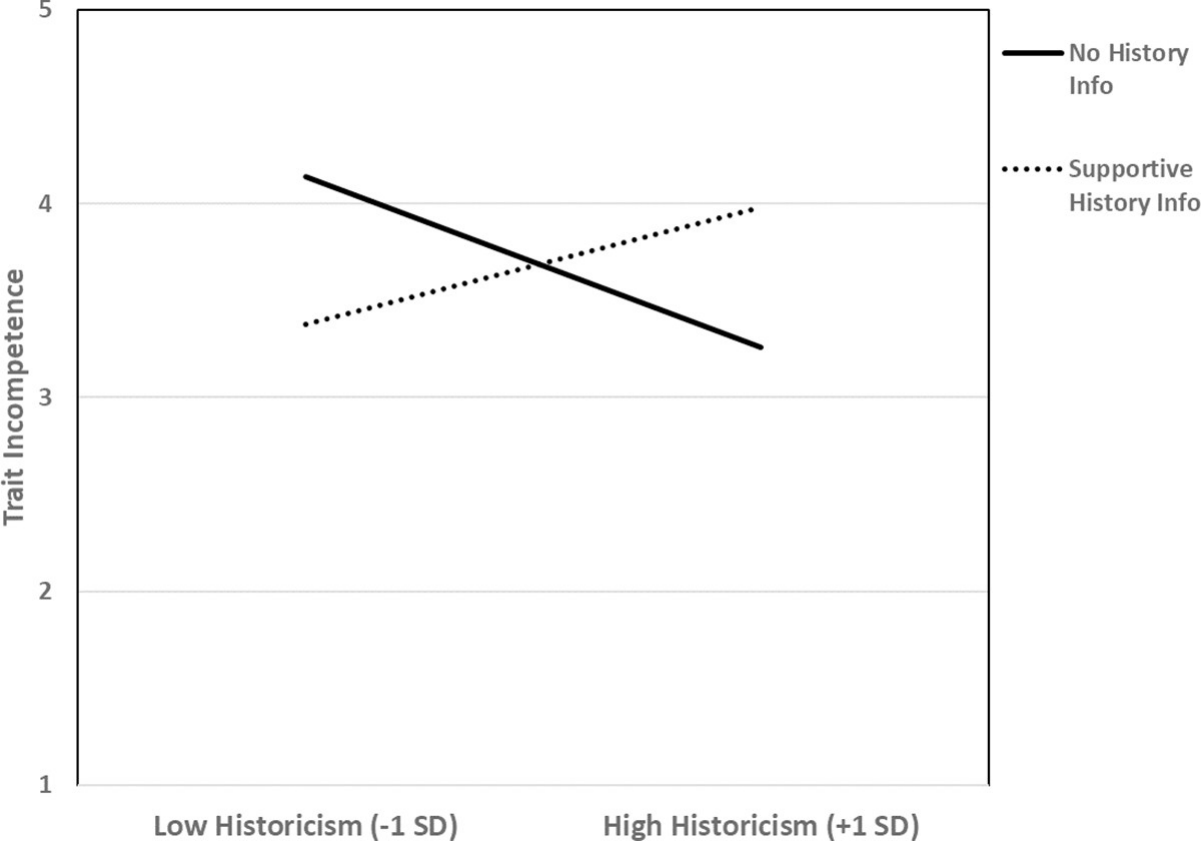
Study 7: Lay historicism is associated with less derogatory trait ratings when no life history information is presented. When life history is described as supportive, however, lay historicism predicts an increase in derogatory trait ratings.

As can also be seen in Table 10, results for our other blame variable—ratings of incompetence—were similar. That is, there was a lay historicism X history information interaction. Simple effects graphed in Fig 7 show that lay historicism predicted reduced character derogation (i.e., blame) in the no history condition, *r*(39) -.33, p = .036, but increased derogation in the supportive history condition, *r*(37) = .35, *p* = .032. In short, these results suggest that lay historicists are less severe in their blame—giving the “benefit of the doubt”—when no historical information is explicitly provided (cf. Study 3), but that this tendency reverses when they learn that the target hails from a supportive environment. Lay historicism does not necessarily lead to “nice” judgments.

Table 10 shows that the lay historicism X history information interaction was quite specific to blame. The only effect of lay historicism outside of blame judgments was, unsurprisingly given the studies above, on compassion. In particular, there was a marginal main effect suggesting that lay historicism was positively associated with compassion for the target, *t*(76) = 1.91, *p* = .059 (*β* = .22). This suggests that, per our argument above regarding blame/compassion dissociation, lay historicists are likely to experience compassion for a struggling target regardless of how much they blame her.

## General discussion

The present work is rooted in attribution theory, one of the most enduring and generative theories in social psychology [11]. Our unique contribution is to propose that everyday people show meaningful variation in a broad lay theory that we call *lay historicism*. Although there is existing work on lay theories [18], we have shown above that these alternative lay theories are unrelated to lay historicism [see also 15]. What is lay historicism? Lay historicists believe that *people’s characteristics are powerfully molded by their life histories*. Based on our findings above involving perceived control of self-formation, it is evident that lay historicism undermines the view that people freely and autonomously create themselves.

Much research has shown that particular attributions—including attributions to life history—can increase compassion and reduce blame. Prior work, however, has involved directly providing participants with concrete history information regarding specific targets: *James is a bully*. *Here is information about his formative years (which provides an explanation for his behavior)*. Thus, although prior work has linked historicist thinking to humane responding [12, 15–17, 31, 53–55], the present article goes beyond prior work to show that concrete history information need not be explicitly provided to generate the prosocial impacts of historicist thinking. This is because some people are *lay historicists* who embrace a *broad*, *abstract historicist theory* which creates a *broad*, *general tendency of humane responding* across different targets and contexts with no history information needing to be explicitly provided. Indeed, we measured compassion in four different ways (Studies 1, 2, 3, 7) and blame in five different ways (Studies 4, 5, 6, 7, implicit blame study in the S2 File) and lay historicism predicted humane responses in every study and did so especially when no history information was explicitly provided (Studies 3, 7).

In all, we presented eight studies that supported a link between lay historicism and humane responding. Several of the studies examined mediating mechanisms. The first three studies focused on compassion. Study 1 showed that lay historicism was positively related to dispositional compassion proneness, mediated by a belief among historicists that past suffering lies behind people’s current struggles. Study 2 showed that lay historicism provides distinct information about compassion proneness, above and beyond the information provided by other well-known predictors of compassion. Finally, Study 3 looked at compassion for a single struggling individual. Results showed that lay historicists imputed a history of suffering for the struggling target when his life history was not explicitly provided, and that this imputed history of suffering predicted increased compassion for him. In short, Studies 1–3 provide consistent evidence that lay historicism is predictive of compassionate responding both as a broad disposition and in specific cases.

Studies 4 through 7—and the implicit blame study in the S2 File—focused on lay historicism and the mitigation of blame. When blame is too intense, it can motivate spiteful mistreatment of the blamed, which can increase future transgressions [56]. Thus, understanding mechanisms of blame mitigation is socially important. Study 4 found that lay historicism was associated with a reduced dispositional tendency to be a harsh blamer, mediated by a belief among lay historicists that individuals do not autonomously forge their own character (i.e., they lack control of self-formation). Study 5 replicated the results of Study 4—including mediation via reduced perceptions of control of self-formation—and reported larger effect sizes. These larger effect sizes were arguably due to the fact that we used a slightly narrower measure of lay historicism that focused on beliefs specifically regarding transgressors. It is also worth noting that in three of our studies lay historicism was assessed many weeks prior to the focal dependent variables, which should assuage concerns that the relation of lay historicism to other variables has been inflated for artifactual reasons (e.g., response biases).

In sum, Studies 1–5 provide support for two basic mechanisms: (1) *Lay Historicism* → *Perceived Suffering* → *Compassion*, and (2) *Lay Historicism* → *(Reduced) Perceived Self-Formative Control* → *(Reduced) Blame*. We also provided evidence suggesting that the perceived suffering mechanism is *specific to compassion* (Study 5) and the control of self-formation mechanism is *specific to blame* (see studies described in the introductory paragraphs to Study 5).

Given the pivotal role of controllability perceptions in blame [11, 14], it is noteworthy that lay historicism is uncorrelated with lay controllability theory. The astute reader will have noticed, however, that the impact of lay historicism on blame mitigation is mediated by diminished perceptions of control of self-formation. Control of self-formation is a type of controllability perception. How, then, can we make sense of this seeming paradox in which historicism is unrelated to lay controllability theory but has effects mediated by control of self-formation? The answer, we believe, is provided by [12]. Those authors show that freedom of action and control of self-formation are distinct beliefs about an agent’s freedom and control. They show, furthermore, that historicism consistently reduces perceived control of self-formation but has virtually no effect on perceived freedom of action. We propose here that lay historicism is unrelated to lay controllability theory because the lay controllability measure primarily taps perceived freedom of action. Indeed, data in Table 5 (and analyses described in the Results section of Study 4) show that the correlation of lay controllability theory with general belief in freedom of action is significantly larger than its correlation with general belief in control of self-formation. Furthermore, regressing lay controllability theory on general belief in freedom of action and general belief in control of self-formation reveals that the former is substantially related to lay controllability theory [*β* = .38; *t*(167) = 4.26, *p* < .001] whereas the latter makes no independent contribution to predicting lay controllability theory [*β* = .04; *t*(167) < 1]. In short, lay historicism is arguably independent of lay controllability theory because lay controllability theory is an indicator of belief in freedom of action, and belief in freedom of action is known to be unrelated to historicism [12].

In Studies 6, 7, and the implicit blame study in S2 File, we went beyond documenting mediational mechanisms. In Study 6, we focused on criminal justice philosophies. We found that lay historicists were significantly more likely than non-historicists to embrace both a philosophy of offender rehabilitation and a philosophy of crime prevention via community investment. These links between lay historicism and humane criminal justice philosophies were independent of participants’ political ideologies and lay controllability theories.

In Study 7, participants reported how much they blamed an irresponsible peer (actually a confederate) following a face-to-face interaction in which the peer disclosed her irresponsible behavior. When no history information was explicitly provided about the peer, lay historicists blamed her less for her irresponsible behavior than did non-historicists. Like Study 3, then, this implies that lay historicists impute exculpatory explanatory histories when such histories are not explicitly provided. Interestingly, the non-blaming stance of lay historicists was flipped in a condition where the peer’s life history was described in terms of a loving, supportive home life (which should foster optimal development). In that condition, lay historicists showed a tendency toward more blame of the peer than non-historicists. This shows that lay historicists are not unfailingly “nice” in their responses. Indeed, they can blame strongly in cases where a positive life history makes the target person appear especially at fault for bad behavior.

One implication of our studies is clear: The proliferation of historicist thinking could contribute to a more humane world. Indeed, we think it is no coincidence that many great leaders of non-violent social movements speak in historicist terms. For example, Archbishop Desmond Tutu utilized historicist arguments as he urged Black South Africans to show mercy to White South Africans (in exchange for White South Africans giving up some political power and expressing the truth about the evils of apartheid): “None of us could predict that if we had been subjected to the same influences, the same conditioning, we would not have turned out like these perpetrators” [57; pg. 85]. Buddhist monk and peace activist Thich Nhat Hahn frequently offers a historicist perspective: “[T]he reasons for a child’s cruelty can be found in his family, society, school, friends, and ancestors. … [In light of this fact], we see that…extreme punishment is not reasonable” [58].

Given our data and these observations about humane leadership, an important question for future research has to do with uncovering the factors that encourage the development of lay historicism. Prior research supports the role of one factor: Deep thinking. Those high in Need for Cognitive Closure are unlikely to become lay historicists [15] and, if they receive historicist information, they are less likely to acknowledge its implications (e.g., perceived suffering) [31]. Although these are important findings, much additional work remains to be done. For example, Study 2 showed that lay historicism is correlated with both perspective taking and identification with humanity. More research is needed to understand the causal links, if any, among these variables.

Research should also examine the types of information exposure that increase lay historicism. For example, there is evidence that education in the social sciences can increase historicist thinking about disadvantaged groups [59, 60]. Does this historicist thinking generalize into a broad, abstract lay historicist theory? How can such generalization be facilitated? Another type of information exposure that should be explored is exposure to books and to visual media. There is evidence that high-quality fiction can improve one’s ability to understand another’s thoughts and feelings [61]. What types of reading might increase development of historicist thinking? What types of T.V. programs or films might play a role?

## Concluding thoughts

The present work provides evidence for meaningful variation in lay historicist theories. The variation is meaningful in the sense that lay historicism predicts increased propensities for compassion and decreased propensities for blame both at the level of broad social dispositions and the level of responses to particular target persons. Lay historicism is a heretofore unexplored contributor to humane responding which, we believe, merits further research attention.

## Supporting information

S1 File Study materials: Scales, dependent measures, vignettes. (DOCX)

S2 File Additional analyses and studies. (DOCX)
