# Supplementary material for: Those who ignore the past are doomed…to be heartless: Lay historicist theory is associated with humane responses to the struggles and transgressions of others
Source: PLoS One. 2021 Feb 19;16(2):e0246882. doi: 10.1371/journal.pone.0246882 (PMC7894831; doi:10.1371/journal.pone.0246882)
Supplement: S1 File — (DOCX) [file pone.0246882.s001.docx]

**THE SOCIAL EXPLANATORY STYLES QUESTIONNAIRE (SESQ; Gill & Andreychik, 2014)**

*Sarah often ridicules and belittles her children. She tells them they are lazy, sloppy, and even "worthless."*

*WHY has Sarah become such a cruel mother?*

***HISTORICISM: *A major factor is Sarah’s prior life experiences, or personal life history.***

1-----------2-----------3-----------4-----------5

NO YES

DISPOSITIONISM: *A major factor is Sarah’s character traits*

1-----------2-----------3-----------4-----------5

NO YES

CONTROLLABILITY: *Sarah has control over her cruelty.*

1-----------2-----------3-----------4-----------5

NO YES

NOTE: The “WHY?” question and explanation text was appropriately changed for each target:

- *Steven never tries to take another’s perspective. When he disagrees with someone, he is stubborn, angry, and insulting.*
- *Janet, a successful accountant, volunteers at a local Food Bank for 6-10 hours per week.*
- *Robert has been arrested numerous times for "petty offenses" since he was 14.*
- *Susan exudes love for others. People look forward to being with her because she shares so much kindness and inspiration.*
- *Bill is very generous with his time and knowledge, patiently helping others even when it is inconvenient for him.*
- *James worked tirelessly to start a number of local Community Centers that provide a variety of activities for community members to enjoy.*
- *Beth sometimes has sexual relations with other men while her husband is out of town.*

**GENERAL BELIEF SCALES from STUDIES 1 & 4**

***Perceived Suffering***

- *A major reason that a person is unkind is that s/he has had much emotional pain in life.*
- *When a parent subjects his/her child to physical or emotional pain, it is typically the case that the parent had suffered similar pain earlier in life.*
- *Some people gossip or tell lies to make themselves look better. It is likely that these people have been hurt by social rejection before and are trying to protect themselves.*
- *If we studied the life history of most criminals, we would find evidence that they suffered from a lot of material and emotional neglect.*
- *A person’s negative character traits are often caused by suffering they have endured in life.*
- *To understand a person’s negative behaviors, one needs to understand the ways in which they have suffered earlier in life.*

***General Belief in Control of Self-formation*** (** = reverse scored)

- *Every person has developed an individual pattern of thinking, feeling, and acting, and that pattern is primarily a product of individual choice.*
- *People forge their own character, individually controlling the type of person they become over time.*
- *People do not freely choose their personality.***
- *Many of our characteristics—behavior patterns, attitudes, values—were never actually chosen by us at all.***
- *By the time we are an adult, we have many personality characteristics that have been instilled in us with little choice or control on our part.***
- *The type of person one grows up to be is substantially out of one’s hands.***
- *Who we are today is generally a product of deliberate self-formation—we each become who we intentionally set out to become.*
- *Each individual freely determines whether she will develop into a nice person or a mean person over the years of her life.*
- *If you meet a kind person, you can be confident that she had control over becoming that kind of person.*
- *For most people, their character traits are something they have freely and deliberately chosen over the course of their lifetime.*
- *If you meet an unfair person, you can be confident that he had control over developing that trait.*
- *Generally speaking, a person with negative character traits never set out to become such a person.***
- *The fact that a person has positive character traits was not entirely up to him.***
- *Our actions and decisions stem from our character traits, which are substantially created by factors over which we have little control.***
- *To a large extent, a person’s nature is determined by forces beyond her control.***
- *Throughout the course of their development—from childhood to adulthood—people have the power to decide what all their personal traits and characteristics will be.*

***General Belief in Freedom of Action*** (** = reverse scored)

- *Human beings have free will: The ability to choose, in every moment, how they will behave.*
- *Using their capacity for free will, people always have the choice to approach a situation with or without compassion.*
- *At all times, a person can freely choose to act badly.*
- *At any moment, a person can freely choose to act morally.*
- *People can always use their capacity for free will to resist negative impulses and enact more positive behaviors.*
- *It is common for a person to be overwhelmed by an irresistible desire that makes her unable to control her actions and make good choices.***
- *When a person has the opportunity to act unfairly, whether she chooses to do so is entirely up to her.*
- *People often lack control over their own behavior.***
- *People generally have strong predispositions that guide their decisions, and it is nearly impossible for them to avoid the influence of those predispositions.***
- *Our behavior involves lots of “automatic habits” over which we have little control.***
- *People can freely choose in all of the decisions they make daily.*
- *People can be “driven” to act in certain ways, with little possibility of choosing to do otherwise.***

***Blame Intensity Inventory (BII; available in Gill & Cerce, 2020)***

**STUDY 3: IMPUTATION OF UNFORTUNATE HISTORIES**

***Deed Only Vignette***

- David is a freshman at college who doesn’t seem to fit in with the rest of the student body. Often he feels rejected or unwanted by his dorm mates and just sits on the sidelines during meal times and other social activities. When his roommate is getting ready to go out at night, David can often be found sitting at his computer surfing the web in his pajamas. Even when his roommate encourages him to join in the fun, David chooses to stay in by himself, electing to remain isolated from others. David appears to put very little effort into making friends. He ignores invitations to social events. He tries to follow a schedule that enables him to avoid other people. Indeed, he comes across as disinterested in others, or even worse, like he just doesn’t like anyone. When people try to engage him in conversation or reach out to him, he does not make much eye contact and does not do his part to contribute to the conversation. His apathetic attitude leaves him friendless.

***Historicist Narrative Vignette***

- Michael is a freshman at college who doesn’t seem to fit in with the rest of the student body. Often he feels rejected or unwanted by his dorm mates and just sits on the sidelines during meal times and other social activities. When his roommate is getting ready to go out at night, Michael can often be found sitting at his computer surfing the web in his pajamas. Even when his roommate encourages him to join in the fun, Michael chooses to stay in by himself, electing to remain isolated from others.
- <HISTORICIST NARRATIVE>: Michael’s social struggles seem to have a lot to do with his experiences growing up in a cold and critical household. From an early age, his parents made him feel socially undesirable and unattractive. Often, his parents simply paid little attention to him. When they did pay attention, however, they tended to be critical of his ideas, his behavior, and his appearance. He left their home with a deep sense of being a generally unlikable person, which has made it hard for him to reach out to others and form social bonds.

***Compassion***

- I feel sympathetic toward David [Michael].
- I feel compassion for David [Michael].

**STUDY 4: LAY HISTORICISM, CONTROL OF SELF-FORMATION, AND BLAME**

**Office Bully Materials Used to Validate Free Will Scales**

**Vignette***: James is the Vice President of a moderately sized company who will often humiliate and harass members of his staff. He says unkind things and implies that employees are unintelligent or incompetent. Employees avoid James as much as possible so that they will not be his next target. Unfortunately, there are few avenues for change in the company as James is the owner's son and employees are hesitant to file complaints against him.*

**Freedom of Action**

- *By using his human capacity for free will, James could choose to STOP being a bully.*
- *It is possible for James to use his free will to overcome his negative habits and behave more appropriately.*
- *Although James may have a strong inclination to treat others poorly, he can use his human capacity for free will to change his behavior.*

**Control of Self-Formation**

- *James had free will in terms of initially BECOMING a bully.*
- *Throughout his life, James was always in control of his personality development.*
- *James’ negative traits are purely a result of him freely choosing to become who he currently is.*

**STUDY 5: HISTORICISM SPECIFICALLY REGARDING TRANSGRESSORS**

***Lay Historicism Regarding Transgressors***

- *When I encounter a hostile and aggressive person, I feel confident that the cause is that he or she grew up in an environment full of hostility and aggression.*
- *A person who is cruel was abused or neglected by others earlier in life.*
- *A person who behaves badly became that way due to an unfortunate or difficult life history.*
- *Life history does NOT have a very powerful effect on moral character development.*
- *Whether one grows up to be a good person or a bad person is only MINIMALLY affected by the types of relationships and experiences one has had in life.*
- *Violent juveniles become violent because they witness and/or are subjected to violence in their homes or neighborhoods.*
- *Moral character is determined by the types of relationships and experiences one has over the course of one's development.*
- *When I see person who lacks compassion for others, I feel confident that he or she did not receive much love and compassion earlier in life.*
- *Prior relationships and experiences--especially during the formative years--determine whether a person develops into a moral or an immoral person.*
- *The quality of the environment in which s/he is raised determines whether a child grows up to be nice or mean.*
- *Moral character develops INDEPENDENT of one's life history.*

*Control of Self-Formation*

- *A bad person has free will in terms of BECOMING that type of person.*
- *Throughout his or her life, a bad person is always in control of his/her personality development.*
- *A bad person's negative traits are purely a result of his/her CHOOSING to acquire those traits.*

*Freedom of Action*

- *By using his/her human capacity for free will, a bad person can choose to STOP behaving badly.*
- *It is possible for an immoral person to choose to behave differently.*
- *Although a bad person might have a strong inclination to treat others poorly, s/he can use the human capacity for free will to act differently.*

***Perceived Suffering***

- *Most criminals have previously experienced a lot of emotional suffering and frustration.*
- *An unkind person has suffered much emotional pain in life.*
- *Behind the offensive words and actions of an immoral person there is much suffering and anguish.*
- *I do NOT think that most bad people have had lives filled with emotional pain and suffering.*

*Blame*

- *I get extremely angry at a person who mistreats others.*
- *I have intense feelings of moral anger towards a person who commits acts of harm.*
- *I feel an urge to lash out at a person who commits acts of violence.*
- *Criminals should be punished harshly for their criminal acts.*
- *When a person is really bad, I get really furious at him or her.*
- *A bad person should be made to suffer for his/her bad deeds.*
- *A bad person does not deserve understanding; s/he deserves punishment.*
- *A person who mistreats others is a sickening, disgusting person.*
- *I despise a person who harms others.*

**STUDY 6: CRIMINAL JUSTICE PHILOSOPHIES**

***Harshness***

- *I am in favor of the death penalty for a person convicted of murder.*
- *There are some cases in which I would think the death penalty is a more appropriate punishment than life in prison with no possibility of parole.*
- *Those who have committed violent acts should be made to suffer because of what they have done; therefore, prisons should not be comfortable places.*
- *In general, I think the criminal justice system needs to deal more harshly with people convicted of crimes.*

***Rehabilitation***

- *Once people are in prison, great effort should be expended to help them transform into law-abiding, good citizens.*
- *To help prisoners develop into productive citizens, vocational training programs (e.g., auto mechanics, electrician training) should be available to them while in prison.*
- *To help them change their ways, prisoners should have access to high school and college-level education while in prison.*
- *Prisoners should have access to psychological services (e.g., counseling) while in prison, to help change the patterns of emotion and behavior that contribute to their illegal acts.*
- *If there is clear evidence that a prisoner has benefitted from education and/or counseling while in prison (i.e., he seems capable of re-entering society as a productive, good citizen), there should be an opportunity for his prison sentence to be shortened.*

***Quarantine:***

- *A crucial function of prisons is to protect society: Dangerous criminals must be kept behind bars.*
- *When a convicted criminal shows no sign of changing his ways, it makes sense to keep him in prison for a longer period of time so that he cannot commit more crimes in the community.*

***Prevention***

- *To fight crime, we would be wise to focus more resources on early intervention programs to help at-risk youths (e.g., youths in communities that produce high numbers of criminals).*
- *It is important to develop and implement treatment programs for abused or neglected children, who are otherwise at great risk for future engagement in criminal behavior.*
- *A major priority should be to invest in ways to prevent kids from taking wrong turns and getting tangled up in gangs, violence, or prison.*

**STUDY 7: FACE-TO-FACE CONVERSATION**

“Irresponsible Student” Script performed by confederate: *I guess I’ve had some difficulties being a good and responsible student. Actually, I feel like I’m pretty forgetful sometimes. A few times, my professors have asked us to turn in our homework, and I was thinking, “What homework?” I completely spaced out on the fact that we had something due! Another time, I was taking part in a group project. The next thing I know, I have 10 texts from the other group members saying, “WHERE ARE YOU?” and “WE ARE MEETING AT LUCY’S CAFE RIGHT NOW.” I totally forgot about our meeting. This kind of stuff has happened even with my friends…for social types of things. Actually, I’ve had friends get angry with me sometimes for forgetting that I was supposed to meet them for lunch, or coffee, or whatever… So, yeah… I guess…a difficulty for me has been trying to be an organized and responsible person.*

“No History” Condition, script performed by confederate: *Well, I guess I like to do lots of the “usual things.” I like to read…there’s some stuff on TV that I like to watch…I like to take walks or hike outside… I really like to hang out with my friends…go out to eat…just talk with them…that kind of stuff… Um, let’s see…what else… I guess that I would like to travel more…I haven’t been very many places…I think I would really enjoy that…maybe someday!*

“Supportive History” Condition, script performed by confederate: *I have a good relationship with my parents. One thing I appreciate is that, before I came to Lehigh, they were very supportive of the things I wanted to do: Studying dance, getting involved with clubs at my school…that kind of stuff. Whenever I had a dance recital, they would be there. My mom was great in terms of helping me keep track of all my activities…she always knew when it was time for dance classes, what days I had certain club meetings…actually, sometimes I wonder if I’d be a little bit more organized if my mom had put a little more responsibility on me <laughs a little bit>. Well…she was just trying to be a good mom…which she is.*

***Magnitude of Struggles***

- *My conversation partner has experienced some difficulties since starting college*

***Blame***

- *My conversation partner’s difficulties are mostly her own fault.*
- *My conversation partner deserves most of the blame for her difficulties.*
- *My conversation partner is the primary cause of her own difficulties.*

***Incompetence***

- *careless, disorganized, lazy, unreliable, conscientious, hardworking, intelligent, reliable*

***Compassion***

- *I felt sympathy for my partner.*
- *I felt sorry for my partner.*
- *I have an understanding attitude toward my partner.*

***Pleasantness During Interview***

- “During this study my conversation partner was”: nice, pleasant, kind, likable, unpleasant, disagreeable, unfriendly, unlikable

***Trait Warmth***

- *sincere, good-natured, warm, pleasant, humorless, moody, unlikable, cold*

***Identification***

- *I can imagine becoming friends with my conversation partner.*
- *My life experiences are similar to my conversation partner’s life experiences.*
- *As a person, I am similar to my conversation partner.*
