# Supplementary material for: Those who ignore the past are doomed…to be heartless: Lay historicist theory is associated with humane responses to the struggles and transgressions of others
Source: PLoS One. 2021 Feb 19;16(2):e0246882. doi: 10.1371/journal.pone.0246882 (PMC7894831; doi:10.1371/journal.pone.0246882)
Supplement: S2 File — (DOCX) [file pone.0246882.s002.docx]

**POWER ANALYSES FOR ALL STUDIES**

Cited sources:

- Kenny D, A. MedPower: An interactive tool for the estimation of power in tests of mediation. 2017. Available from: https://davidakenny.shinyapps.io/MedPower/.
- Faul F, Erdfelder E, Lang A, Buchner A. G* Power 3: A flexible statistical power analysis program for the *social, behavioral, and biomedical sciences. Behavior research methods 2007;39(2):175-191.*
- Perugini M, Gallucci M, Constantini G. A practical primer to power analysis for simple experimental designs. International Review of Social Psychology 2018(31(1)):1-23.

Study 1: We used Kenny’s (2017) MedPower to compute a power analysis. Pilot work in our lab suggested that we could reasonably expect lay historicism to predict perceived suffering with a standardized effect size of *β* = .35. Results in [31] suggest that perceived suffering is strongly related to compassion, and thus that a standardized effect size of *β* = .40 would be a conservative expectation. Assuming these two effect sizes, MedPower determined that we need a sample size of *N* = 71 to achieve 80% power to detect the resulting indirect effect (*α* = .05; two-tailed).

Study 2: We used G*Power to compute a power analysis. Assuming that lay historicism will explain 4% of the variance in compassion (as it did in Study 1) and that our entire set of predictor variables—most of which are well-known to be strong predictors of compassion—will explain 30% of the variance in compassion, G*Power determined that we would need a sample size of *N* = 140 to achieve 80% power (*α* = .05; two-tailed) to detect a significant effect of lay historicism in our overall regression model with six predictors (i.e., lay historicism, perspective taking, identification with humanity, biological sex, implicit person theories, lay controllability theories).

Study 3: We computed statistical power using G*POWER. Our key prediction was an interaction between lay historicism and an experimental manipulation of the presence vs. absence of information about a target individual’s unfortunate history. Our manipulation of history information in Study 3 is within-participants. This means that our hypothesis concerns the difference between two dependent correlations (i.e., lay historicism with perceived suffering in the no history condition; lay historicism with perceived suffering in the history condition). To compute power, we assumed that the effect size for the lay historicism-perceived suffering correlation would be equal to the observed correlation in Study 1 (*r* = .40). We assumed that this correlation would be eliminated when an explicit historicist narrative is provided (*r* = .00). Finally, we assumed that the correlation between ratings of perceived suffering in the no history and history conditions would be *r* = .50. Based on these assumptions, G*Power determined that we would need a sample size of *N* = 38 to achieve 80% power to detect our predicted difference.

Study 4: We used Kenny’s (2017) MedPower to compute a power analysis. Pilot work in our lab suggested that we could reasonably expect lay historicism to predict perceived control of self-formation with a standardized effect size of *β* = -.25. Previous work suggests that perceived control of self-formation is strongly related to blame, and thus that a standardized effect size of *β* = .40 would be a reasonable expectation [12]. Assuming these two effect sizes, MedPower determined that we need a sample size of *N* = 123 to achieve 80% power to detect the resulting indirect effect (α = .05; two-tailed).

Study 5: Same analysis as Study 4 is applicable.

Study 6: We planned to compute correlations between lay historicism and the different dimensions of criminal justice philosophy. We wanted to be able to detect small correlations (r = .20). A power analysis using G*Power indicated that a sample size of *N* = 193 would provide 80% power to detect *r* = .20 (α = .05, two-tailed).

Study 7: Our key prediction was an interaction between lay historicist theories and a manipulation of the target individual’s life history information (i.e., none vs. supportive history). We expect lay historicist theories to be associated with blame reduction in the no history condition (cf. Study 3) but with blame aggravation in the supportive history condition. Prior work on lay historicism and blame of those with controllable shortcomings [15, Study 6] suggests that we can expect an effect size of *r* = -.30 in the no history condition. Because our prediction of an effect reversal in the supportive history condition is novel—and because a null effect is also a reasonable prediction there—we will conservatively assume an effect size of *r* = .15 in that condition. Based on these assumptions about effect size, we used procedures described in Perugini et al. (2018) to compute power for a 2-way interaction between a categorical and continuous variable. We determined that we need a sample size of *N* = 68 to achieve 80% power to detect our predicted interaction (*α* = .05).

Implicit Blame study (in Supplemental Materials below): We did not do a prior power analysis and instead simply ran as many participants as we could in a single semester. In this study, we discovered two effects of interest: (A) A two-way interaction with effect size of *f^2^* = .294 [computed by G*Power based on our inputs of variance explained by the interaction—i.e., 10.2%--and residual variance unexplained by the predictors—i.e., 34.7%)], and (B) A follow-up simple effect (relation between lay historicism and implicit blame) with an effect size of *f^2^* = .343 [computed as just described]. According to G*Power’s post hoc power computation, our sample size of *N* = 59 provided greater than 98% power to detect these effects.

**STUDY 4: Mediation of the effect of lay controllability theories on harsh blame**

General belief: Freedom of Action

.07

.48***

Harsh Blame

Lay controllability theory

.21*

.34***

General belief: Control of Self-Formation

Indirect effect through freedom of action: .03 (95% CI: -.07, .14)

Indirect effect through control of self-formation: .07 (95% CI: .004, .19)

**CORRELATIONAL ANALYSES SEPARATING LAY HISTORICISM FOR**

**POSITIVE VS. NEGATIVE BEHAVIORS** (***p < .001; **p < .01; *p<.05; †p < .08)

| **STUDY 1** | Perceived Suffering | Compassionate Love |
| --- | --- | --- |
| SESQ Historicism – Positive Behaviors | .30*** | .17* |
| SESQ Historicism – Negative Behaviors | .44*** | .20* |
| **Significance of diff between *r*s** | *z* = 2.05, *p* = .04 | *z* = .41, *p* = .68 |
| ***r*(149) = .58, *p* < .001 for pos and neg** |  |  |

| **STUDY 2 (.67***)** | Empathic Concern |
| --- | --- |
| SESQ Historicism – Positive Behaviors | .23** |
| SESQ Historicism – Negative Behaviors | .34*** |
| **Significance of diff between *r*s** | *z* = 1.83, *p* = .07 |
| ***r*(166) = .67, *p* < .001 for pos and neg** |  |

| **STUDY 3** | Perceived Suffering – No history | Perceived Suffering – Historicist Narrative | Compassion – No History | Compassion – Historicist Narrative |
| --- | --- | --- | --- | --- |
| SESQ Historicism – Pos | .22† | .06 | .29* | .23† |
| SESQ Historicism – Neg | .34** | .16 | .31* | .37** |
| **Significance of diff between *r*s** | *z* = 1.37, *p* = .17 | *z* = 1.10, *p* = .27 | *z* = .23, *p* = .82 | *z* = 1.62, *p* = .11 |
| ***r*(65) = .73, *p* < .001 for pos and neg** |  |  |  |  |

| **STUDY 4** | Control of Self-Formation | Freedom of Action | Blame Intensity |
| --- | --- | --- | --- |
| SESQ Historicism – Pos | -.09 | -.09 | .11 |
| SESQ Historicism – Neg | -.22** | -.21** | .02 |
| **Significance of diff between *r*s** | *z* = 2.03, *p* = .04 | *z* = 1.87, *p* = .06 | *z* = 1.38, *p* = .17 |
| ***r*(170) = .64, *p* < .001 for pos and neg** |  |  |  |

**STUDY 5 – N.B.: Study 5 only included negative behaviors when assessing lay historicism.**

**STUDY 6 – N.B.: At some unknown time point, the individual items from the SESQ were deleted from this file and thus separate subscale scores cannot be computed.**

| **STUDY 7** | Blame | Incompetence | Compassion |
| --- | --- | --- | --- |
| SESQ Historicism – Pos (*r*) | -.09 | .00 | .21† |
| SESQ Historicism – Neg (*r*) | -.09 | .01 | .26* |
|  |  |  |  |
| **Regression** |  |  |  |
| **Positive behavior historicism** | -.10 | -.01 | .21† |
| History condition | -.02 | -.01 | .10 |
| Pos Historicism X condition | .20† | .26* | -.16 |
|  |  |  |  |
| **Regression** |  |  |  |
| **Negative behavior historicism** | -.07 | .06 | .25* |
| History condition | -.03 | -.01 | .11 |
| Neg Historicism X condition | .16 | .31** | -.07 |
| ***r*(78) = .51, *p* < .001 for pos and neg** |  |  |  |

**Note. The r and β values here are so similar that a statistical test seems unnecessary.**

**IMPLICIT BLAME STUDY**

In Studies 1 through 7 in the body of our article, the dependent variables were explicit reports. Does lay historicism affect only such explicit responses, or does it also have an impact on implicit responses? Prior work has documented links between historicist thinking and implicit attitudes toward an outgroup (Andreychik & Gill, 2012). Those studies, however, concerned cases where the historicist thoughts were specifically about the outgroup under consideration. Here, we examine whether the broad, abstract lay historicist theory is associated with mitigation of implicit blame of a specific transgressor.

**Method**

**Participants*.*** Fifty-nine undergraduate students (28 female) participated for course credit. Sample size was determined by the number of participants we were able to run in one semester.

**Procedure*.*** At a pretest, participants completed our measures of *lay historicism* and *lay controllability theory*. Several weeks later, they reported individually to the lab for an ostensibly unrelated study. They sat at a computer in a private cubicle and received instructions via the computer. They learned that they would complete several tasks. First, they would read about a person. Later, they would take a memory test on that information. Participants also learned that, in between reading about the person and the memory test, they would be presented with a “multi-tasking” activity to be described later.

After receiving these instructions, participants read a short vignette about a transgressor:

*Robert likes to intimidate people. He has no desire to help anyone else but himself, and he thinks of crime as a great way to get easy money. At an early age, Robert began committing crimes, feeling no remorse for his victims. Committing crimes makes him feel powerful and important. Robert committed his first assault and robbery as a teenager. Since then, he has routinely threatened people with a knife, punched and kicked them, and broken into their homes to steal their money and valuables.*

They were reminded that a memory test would come later, after the multi-tasking activity. The multi-tasking activity was actually an assessment of implicit blame. Implicit blame was indexed by the accessibility of blame-relevant words following priming with the transgressor’s name (*Robert*).

After the vignette, the computer program provided instructions for the implicit blame (“multi-tasking”) task. Participants learned that they would see a variety of male and female names and that, later, they would be asked to discriminate names they had seen from names they had not seen. Interspersed with the name task, they learned, will be a “word/non-word” task (i.e., a lexical decision task). That is, they learned that a string of letters would be presented immediately following each name, and that sometimes the string will be an actual English word (e.g., *house*) but other times it will not (e.g., *hoseuat*). Participants were instructed to press the “d” key if the string of letters is a word and the “k” key if it is a non-word. They were also instructed to keep their left and right index fingers on those keys during the entire task. Participants completed 12 practice trials to familiarize them with the task.

The implicit blame task consisted of 100 randomized trials. On each trial, a fixation point—a row of X’s—was presented in the center of the screen for 1000 ms. Then, a name was displayed for 1000 ms. Next, another row of X’s appeared for 250 ms, followed by a letter string. The string of letters remained on the screen for 250 ms. It was followed by a blank screen which remained until participants indicated whether the string was a word or a non-word. After that, a blank screen was displayed for 500 ms and then the next trial began. The computer recorded responses and response times (RTs) for each trial.

Critical trials involved the transgressor’s name (*Robert*) followed by one of three blame-related words: *Blame*, *anger*, and *bad*. Each blame word appeared twice following the transgressor’s name, so there were six trials that tapped response times to blame words following the transgressor’s name. Furthermore, the transgressor’s name was followed by neutral words (*ceramic*, *sentence*, *example*) on three trials and by non-words on three trials. Thus, there were twelve trials involving the transgressor’s name. To control for individual differences in the accessibility of blame words, we included twelve trials with the name *Steven* which had the same arrangement of words and non-words as in the *Robert* trials (also in randomized order). The names *Steven* and *Robert* have been shown to receive highly similar warmth and competence ratings (Newman et al., 2018), and thus Steven is an appropriate control name. The remaining seventy-six trials were “filler” trials using a mix of male and female names paired equally often with neutral words and non-words. Blame-related words were not used in these filler trials.

For each of the nine transgressor (*Robert*) and nine control (*Steven*) trials that involved words (rather than non-words), we deleted any RTs that were greater than |3 *SD*| from the trial mean or that were associated with errors (i.e., indicating “non-word” following a word). This resulted in the deletion of 2.39% of the RTs. As our index of *implicit blame*, we computed the average RT in milliseconds for the six trials on which blame-relevant words followed the transgressor’s name. To control for individual differences in RTs, we computed the average RT for trials on which neutral words followed the transgressor’s name. Finally, to control for individual differences in the accessibility of blame concepts, we computed the average RT for trials on which blame-relevant words followed the control name. For the implicit blame index, low scores—faster RTs—are indicative of more implicit blame (i.e., greater accessibility of blame-related words). For ease of interpretability, we recoded the data so that high scores would be indicative of more implicit blame (and we recoded the other RT variables, too, for consistency). Specifically, for each of the three RT indexes just described, we subtracted each participant’s score from the mean and then added the mean back to that result. Following this transformation, a score that was, say, 50 ms above the mean becomes a score that is 50 ms below the mean.

**Results and Discussion**

Descriptive statistics and correlations among variables are in the table below:

|  | *M*  [possible range] | *SD* | α | 1. | 2. | 3. | 4. |
| --- | --- | --- | --- | --- | --- | --- | --- |
| 1. Lay Historicism | 4.08  [1-5] | .60 | .80 |  |  |  |  |
| 2. Lay Controllability Theory | 4.21  [1-5] | .68 | .88 | -.01 |  |  |  |
| 3. RT to blame words following priming  with transgressor’s name | 589 ms  [364-779] | 98 | .79 | -.11 | .14 |  |  |
| 4. RT to blame words following priming  with control name | 579 ms  [270-730] | 93 | .72 | -.04 | .25† | .76*** |  |
| 5. RT to neutral words following priming  with transgressor’s name | 626 ms  [357-830] | 104 | .47 | .09 | .32* | .49*** | .46*** |

*Note*. †*p* = .054, **p* < .05, ***p* < .01, ****p* < .001

To examine the link between lay historicism and implicit blame, we computed a multiple regression analysis. As noted in the Introduction, for all studies we included the effect of lay controllability theories and the interaction between lay historicism and lay controllability theories in our preliminary analyses. We present significant results from those analyses in the relevant Results sections. Here, we have a significant effect to report. We regressed implicit blame of the transgressor on lay historicism (mean-centered), lay controllability theories (mean-centered), and the lay historicism X lay controllability theories interaction. The analysis controlled for accessibility of blame-related words following the control prime (i.e., *Steven*) and for blame-irrelevant individual differences in RTs (i.e., RTs to neutral words following the transgressor’s name). Results are presented in Table 12. As can be seen there, both control variables were significantly and positively related to implicit blame. More importantly for present purposes, we found that lay historicism had a marginal negative relation with implicit blame and, furthermore, that the effect of lay historicism on implicit blame was moderated by lay controllability theory. We note that this interaction was robust to various treatments of our data. For example, the interaction remains if we analyze log transformed reaction times to correct for skew, *t*(53) = 2.43, *p* = .019 (β = .21). It remains if we remove the control variable of RTs for neutral words, *t*(53) = 2.29, *p* = .026 (β = .20). It also remains—albeit marginal—if we simply analyze raw reaction time without excluding outliers, *t*(53) = 1.77, *p* = .083 (β = .19). The only control variable whose removal obscures the interaction effect—reducing it to *t*(53) = 1.16, *p* = .25 (β = .14)—is RTs for blame-relevant words following the control name. This suggests that it is crucial to control for the fact that, even following a neutral prime, blame words are highly accessible to some people and not especially accessible to others.

**Multiple regression: Lay Historicism and Implicit Blame**

|  | β | *t*(53) | *p* |
| --- | --- | --- | --- |
| RT: blame words following neutral prime | **.70***** | **7.53** | **< .001** |
| RT: neutral words following transgressor prime | **.22*** | **2.28** | **.027** |
| Lay Historicism | **-.15†** | **-1.81** | **.076** |
| Lay Controllability Theory | -.05 | -.53 | .596 |
| Lay Historicism X Lay Controllability Theory | **.22*** | **2.55** | **.014** |

*Note.* High scores indicate more implicit blame. Significant and marginal effects are bolded.

The pattern of the interaction can be seen in this figure:


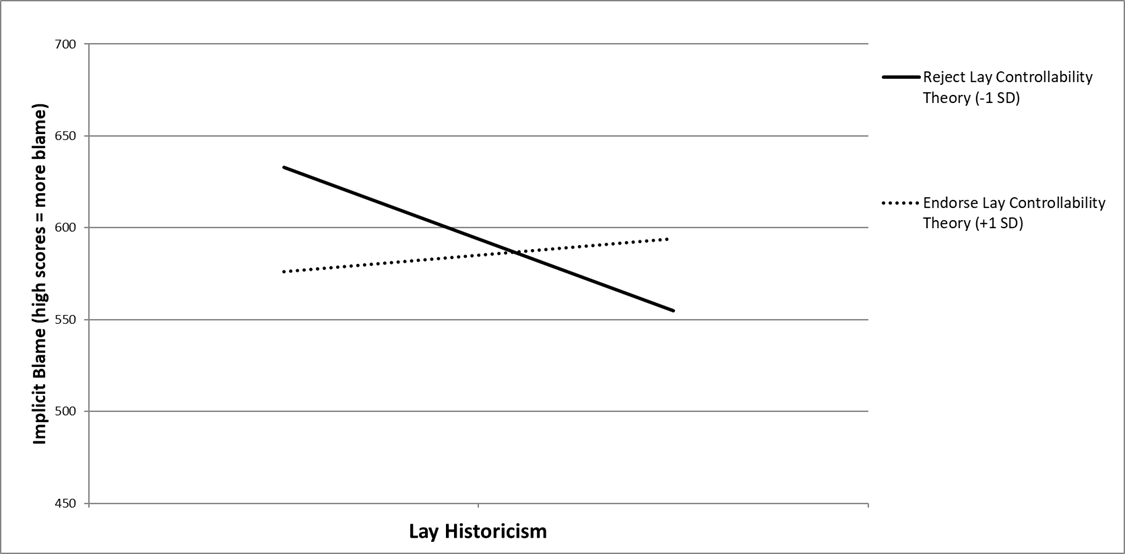


As can be seen there, among those who reject lay theories of controllability, lay historicism had a significant negative relation to implicit blame, *t*(53) = -2.79, p = .007 (β = -.40). This shows that the tempered blame responses of lay historicists (see Studies 4, 5, and 7) can also be detected by implicit measures. In contrast, among those who endorse the lay theory of controllability, lay historicism was unrelated to implicit blame, *t*(53) = .83, *p* = .41 (β = .09).

This result suggests that lay historicism does foster blame mitigation even on implicit measures, but the phenomenon is a bit more complex than in the studies of explicit judgments above. To mitigate implicit blame, lay historicism must exist alongside a general rejection of the lay theory of controllability. This finding reminds us of recent work by Gill and Ungson (2018). They used mouse tracking data to demonstrate that the tempered blame elicited by a historicist narrative involved a high level of uncertainty (i.e., non-linear mouse trajectories when making blame judgments via mouse clicks). If we assume that blame uncertainty will be reflected in continued indication of blame on implicit measures (i.e., the implicit measure taps the lingering sense that strong blame might be appropriate), then the present results could mean that the lay historicist theory contributes to relatively uncertain blame mitigation (i.e., implicit blame remains) if the historicist has a strong belief that people are generally in control of their actions, but the lay historicist theory contributes to certain blame mitigation (i.e., implicit blame is removed) when the historicist has a strong belief that people are generally not in control of their actions. This reasoning fits with the finding from Gill and Ungson that blame uncertainty stems from uncertainty about how much control the transgressor truly had over his transgressing (i.e., *Did his difficult life really deprive him of control?*).

- Filler names for implicit blame task: PHILLIP, JEAN, MICHELLE, GENE, ASHLEY, REBECCA, JENNIFER, PATRICK, ANDREW, MEGAN, RICHARD, WILLIAM

Cited work:

Andreychik MR, Gill MJ. Do negative implicit associations indicate negative attitudes? Social explanations moderate whether ostensible “negative” associations are prejudice-based or empathy-based. J Exp Soc Psychol 2012;48(5):1082-1093.

Gill MJ, Ungson ND. How much blame does he truly deserve? Historicist narratives engender uncertainty about blameworthiness, facilitating motivated cognition in moral judgment. Journal of Experimental Social Psychology 2018; 77:11-23.

Newman LS, Tan M, Caldwell TL, Duff KJ, Winer ES. Name norms: A guide to casting your next experiment. Person Soc Psychol Bull 2018;44(10):1435-1448.
